# Chilean Rhubarb, *Gunnera tinctoria* (Molina) Mirb. (Gunneraceae): UHPLC-ESI-Orbitrap-MS Profiling of Aqueous Extract and its Anti-*Helicobacter pylori* Activity

**DOI:** 10.3389/fphar.2020.583961

**Published:** 2021-01-27

**Authors:** Sonja Hebel-Gerber, Apolinaria García-Cancino, Angélica Urbina, Mario J. Simirgiotis, Javier Echeverría, Luis Bustamante-Salazar, Katia Sáez-Carrillo, Julio Alarcón, Edgar Pastene-Navarrete

**Affiliations:** ^1^ Laboratorio de Farmacognosia, Departamento de Farmacia, Facultad de Farmacia, Universidad de Concepción, Concepción, Chile; ^2^ Laboratorio de Patogenicidad Bacteriana, Departamento de Microbiología, Facultad de Ciencias Biológicas, Universidad de Concepción, Concepción, Chile; ^3^ Departamento de Producción Vegetal, Facultad de Agronomía, Universidad de Concepción, Chillán, Chile; ^4^ Instituto de Farmacia, Facultad de Ciencias, Universidad Austral de Chile, Campus Isla Teja, Valdivia, Chile; ^5^ Departamento de Ciencias del Ambiente, Facultad de Química y Biología, Universidad de Santiago de Chile, Santiago, Chile; ^6^ Departamento de Análisis Instrumental, Facultad de Farmacia, Universidad de Concepción, Concepción, Chile; ^7^ Facultad de Ciencias Físicas y Matemáticas, Universidad de Concepción, Concepción, Chile; ^8^ Laboratorio de Síntesis y Biotransformación de Productos Naturales, Departamento de Ciencias Básicas, Universidad de Bío-Bío, Chillán, Chile

**Keywords:** Mapuche food, Metabolomics, Gunnera tinctoria, Nalca, Centrifugal Partition Chromatography, HPLC-MS Orbitrap, ulcer, *Helicobacter pylori*

## Abstract

The full UHPLC-MS metabolome fingerprinting and anti-*Helicobacter pylori* effect of *Gunnera tinctoria* (Molina) Mirb. (Nalca) total extract (GTE) and fractions prepared from its edible fresh petioles were evaluated. The activity of *G. tinctoria* against *H. pylori* strains ATCC 45504 and J99 was assessed *in vitro* by means of agar diffusion assay, Minimum Inhibition Concentration (MIC), and Minimum Bactericidal Concentration (MBC), while killing curve and transmission electronic microscopy (TEM) were conducted in order to determine the effect of the plant extract on bacterial growth and ultrastructure. Additionally, the inhibitory effect upon urease was evaluated using both the Jack Bean and *H. pylori* enzymes. To determine which molecules could be responsible for the antibacterial effects, tentative identification was done by ultra-high performance liquid chromatography coupled with high-resolution mass spectrometry (UHPLC-Q-Orbitrap®-HR-MS). Furthermore, the total *G. tinctoria* extract was fractionated using centrifugal partition chromatography (CPC), giving four active fractions (1–4). It was determined that the crude extract and centrifugal partition chromatography fractions of *G. tinctoria* have a bactericidal effect being the lowest MIC and MBC = 32 μg/ml. In the killing curves, fraction one acts faster than control amoxicillin. In the urease assay, F3 exhibited the lowest IC_50_ value of 13.5 μg/ml. Transmission electronic microscopy showed that crude *G. tinctoria* extract promotes disruption and separation of the cellular wall and outer membrane detachment on *H. pylori* causing bacterial cell death.

## Introduction

**FIGURE 1:**
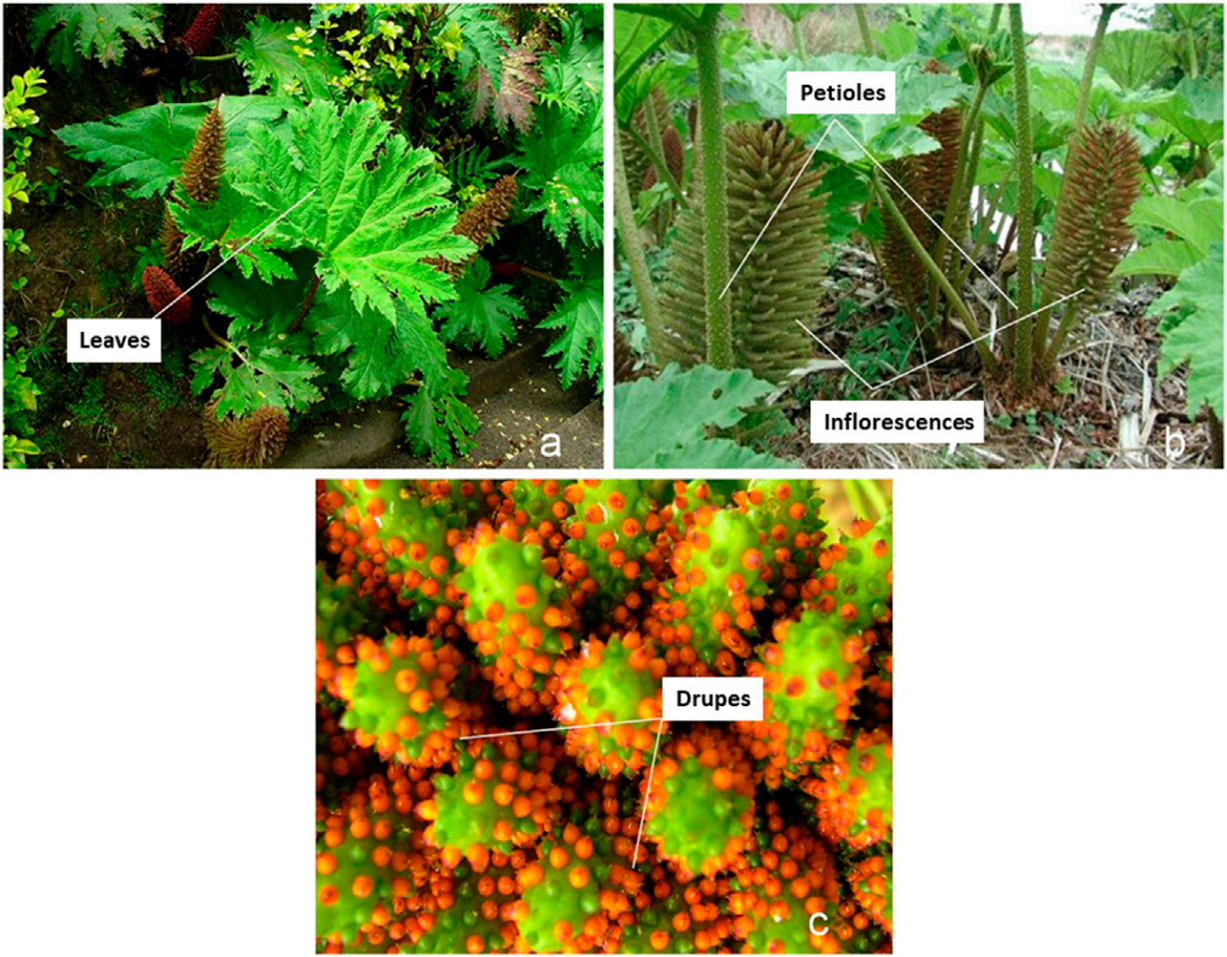
Pictures of *Gunnera tinctoria* taken in April 2018 in *Lebu*, *Región del BíoBío*. **(A)** Leaves, **(B)** petioles and inflorescences, and **(C)** close-up of drupes.

*Gunnera tinctoria* (Molina) Mirb. (*Gunneraceae*, Figure 1) is a medicinal and edible plant also known as *Pangue*, Nalca (Mapuche voices), or Giant Chilean Rhubarb. The *Gunnera* genus has wide geographical distribution in South America and twelve species can be found in Chile. *G. tinctoria* was described for the first time by the South American botanist Juan Ignacio Molina in 1782. In folk medicine, the plant is used as a hemostatic, astringent, and febrifuge (Muñoz et al., 2004). Furthermore, the stems (petioles) are eaten raw, being sold in the streets and from local herbalists in the central south of Chile. On the other hand, *H. pylori* is a microaerophilic Gram-negative bacterium with a great ability to colonize human gastric mucosa. It has a 50% prevalence worldwide (Go, 2002; Malfertheiner et al., 2012) and 73% prevalence in Chile (Ferreccio et al., 2007). *H. pylori* infects the gastric epithelium (Wroblewski et al., 2010; Skoog et al., 2012) and its presence is related to gastric pathologies like gastritis, MALT lymphoma, and peptic ulcer (Go, 2002; Peek and Crabtree, 2006; Sachs and Scott, 2012). Importantly, due to its clear relation with gastric cancer, in the year 1994, the IARC defined *H. pylori* as a Group I human carcinogen (Parkin et al., 2005). These pathogenic features are in part due to its bacillary and curved s-shape (spiral) form plus several flagella that confer with its high mobility. Also, *H. pylori* possesses oxidase, catalase, urease, and carbonic anhydrase (Mobley et al., 1991; Takeuchi et al., 2008). These enzymes help to neutralize the acidic environment of the stomach, allowing *H. pylori* to survive for decades in the gastric epithelium (Wroblewski et al., 2010; Skoog et al., 2012). Hitherto, there are no available vaccines, antibodies, or oligosaccharides that could be used successfully against the bacteria, blocking its adherence to the gastric mucosa. The current pharmacological regime for eradication currently combines two antibiotics (clarithromycin plus amoxicillin or metronidazole) and a proton pump inhibitor such as omeprazole (Malfertheiner et al., 2017). This therapy is expensive and can last for 10–14 days. This situation increases the incidence of side effects and many times produces dropout in the therapy. The final consequence of dropping out from antibiotic therapy is the failure to eradicate the infection in 10–30% of patients (Bohr and Malfertheiner, 2009; O’Connor et al., 2014). Polyphenol-rich extracts have been investigated in many natural products regarding their anti-*H. pylori* effects (González-Segovia et al., 2008; Wang, 2014). Phenolic compounds not only can promote *H. pylori* death but also could neutralize certain virulence factors and reduce the inflammatory process. For instance, Ruggiero and coworkers (Ruggiero et al., 2007) reported that the most representative polyphenol found in green tea and wine is able to reduce the gastric injury induced by both *H. pylori* and its VacA-purified toxin. Additionally, in our previous work, we reported the importance of catechin-derived procyanidins extracted from *Peumus boldus* leaf extracts and its antiurease and antiadhesive properties assessed in AGS cells (Pastene et al., 2014). According to literature, *G. tinctoria* branches and leaves possess triterpenes and sterols such as β-sitosterol, daucosterol, pinoresinol, oleanolic acid, erythrodiol, uvaol, lupeol, and vomifoliol (Muñoz et al., 2004). Interestingly, even though petioles of this plant have been consumed for centuries, there is a lack of available chemical and pharmacological data. A recent paper (Zamorano et al., 2017) reported some antioxidant effects and phenolic compounds of *G. tinctoria*; however, no mass spectrometry analysis was reported to give an accurate idea of the phenolic constituents in the plant. Another recent investigation reported *in vitro* antimicrobial effects of *G. tinctoria* extracts prepared from flowers, roots, and petiole botanical parts (Velásquez et al., 2020). In particular, petiole *G. tinctoria* extracts exhibited a significant effect against *Pseudomonas aeruginosa*, *Staphylococcus aureus,* and *Escherichia coli* with a MIC value of 4.7 mg/ml. Albeit UHPLC was carried out, the analytical data reported by these authors refer only to the presence of caffeic, coumaric, and gallic acids in all the botanical parts and quercetin and rutin only in the petioles. In the present paper, we have studied the potential of this medicinal and edible plant as a source of antimicrobial molecules. Moreover, we reported for the first time the complete metabolome profile using high-resolution Q-Orbitrap technology coupled to a PDA detector (UHPLC-PDA-Q-Orbitrap-HR-MS), and we demonstrate that *G. tinctoria* aqueous extract and their fractions obtained by CPC have *in vitro* lethal effect against the important pathogen *H. pylori*.

## Material and Methods

### Chemicals and Reagents

UHPLC-MS grade solvents, formic acid, and LC-MS were from Merck (Santiago, Chile). Ultrapure water was obtained from a water system of purification (Milli-Q Merck Millipore, Chile)*.* Jack Bean urease was purchased from Sigma-Aldrich (Santiago, Chile). Flavonol and anthocyanin standards (ellagic acid, rutin, quercetin, kaempferol, cyanidin, and cyanidin-3-*O*-glucoside, all standards with a high purity: 95% by HPLC) were acquired from ChromaDex (Santa Ana, CA, USA), Sigma-Aldrich (Saint Louis, Mo, USA), or Extrasynthèse (Genay, France).

### Plant Material and Extraction

*G. tinctoria* petioles (1.5 kg) were purchased in the local market of Concepción, Chile. This plant sample was collected in Lebu, Provincia de Arauco, Región del Biobío, Chile (-37°41'21.0"S 73°38'50.6"W). The sample was identified by Dr Alicia Marticorena from Departamento de Botánica de la Facultad de Ciencias Naturales y Oceaonográficas de la Universidad de Concepción (UDEC). A voucher specimen (CONC180716) has been preserved in the aforementioned UDEC herbarium. Fresh petioles were peeled and cut in cylindrical portions from 5 to 10 cm and stored at −20 °C until use. Three kg of a pool of frozen *G. tinctoria* petioles was thawed with distilled boiling water, chopped into pieces of 5 × 5 mm, and introduced again in the water. Then, the petioles were shaken at 300 rpm for 30 min and filtered through cotton, and the collected liquid was saved. The procedure was repeated using the same vegetable sample but with 95% ethanol water. Clarified liquids were mixed and diluted with distilled water until the alcohol grade reached around 10%. The water content of fresh petioles was determined by drying in a heating oven at 105 °C for 48 h. The sample was weighed every 24 h until a constant weight was achieved.

### Preparation of *G. tinctoria* Extract Through Sepabeads SP-850

Aqueous extracts were adsorbed into columns (40 × 3 cm) filled with the macroporous resin Sepabeads SP-850 preconditioned with water. After loading the sample, the column was washed with water to eliminate sugars, salts, and proteins, and then adsorbed compounds were recovered with 95% ethanol. The ethanol extract was concentrated under vacuum and finally freeze-dried. *G. tinctoria* total extract (GTE, 27.0 g) was stored at −20 °C until use.

### Fractionation of *Gunnera tinctoria* (Molina) Mirb. (Nalca) Total Extract by Centrifugal Partition Chromatography

Fractionation of GTE was performed using a Spot-CPC-250-B Bio-Extractor (CPC, Armen, France) in the elution-extrusion mode. The system has a four-way switching valve that allows operation in either the descending or ascending modes. The CPC unit was connected to a SPOT-PREP-II system (Armen, France), with an integrated UV detector and fraction collector. CPC separation was performed with a two-phase solvent system composed of MtBE-BuOH-ACN-H_2_O with 0.001%TFA at 4:2:3:8 v/v (Hubert et al., 2013). The solvent mixture was automatically generated by the SPOT-PREP-II unit. The CPC rotor was first filled with 1.5 column volumes using the lower aqueous phase at 30 ml/min and 500 rpm rotation. The upper phase was then pumped into the system in the ascending mode at a flow rate of 10 ml/min and rotation was increased from 500 to 2000 rpm. After equilibrium was reached, the samples (2 g of GTE) were dissolved in 10 ml 1 : 1 mixture of upper and lower layers and injected into the CPC system. The running time in ascending mode was 65 min. The extrusion step was carried out with the polar stationary phase (lower) at 15 ml/min and 1800 rpm for 25 min. Elution was monitored using scan 200–600, 280, 350, and 520 nm wavelengths, collecting fractions of 25 ml. Fractions with similar composition were reunited according to online UV spectra and thin layer chromatography analysis.

### UHPLC-DAD-MS Instrument

The Dionex Thermo Scientific Ultimate 3000 UHPLC system connected with a Thermo Q Exactive Focus machine was used as previously informed (Simirgiotis et al., 2016b). For the analysis, the extracts were redissolved (2 mg/ml) in ethanol-distilled water (1:1 v/v) and 10 μL of filtered solution (PTFE filter) was injected into the instrument, with the specifications set as already informed (Simirgiotis et al., 2016b).

### LC Parameters and MS Parameters

Liquid chromatography was performed using an Acclaim UHPLC C18 column (Acclaim, 150 mm × 4.6 mm ID, 2.5 μm, Thermo Fisher Scientific, Bremen, Germany) set at 25°C. The wavelengths detected were 354, 254, 280, and 330 nm, and DAD was acquired from 200 to 800 nm for full characterization of peaks. Mobile phases employed were acetonitrile (B) and 1% aqueous formic solution (A) while the gradient program was 0.00 min, 7% B; 5.00 min, 7% B; 10.00 min, 25% B; 15.00 min, 33% B; 20.00 min, 85% B; 25.00 min, 90% B; 35.00 min, 7% B; and 15 min for column equilibration before injections. The flow rate employed was 1.00 ml min^−1^, and the injection volume was 10 μL. Standards and the resin extract dissolved in methanol were maintained at 10 °C during storage in the autosampler. The HESI II and Orbitrap spectrometer parameters were set as informed previously (Simirgiotis et al., 2016c; Qi et al., 2017).

### *Helicobacter pylori* Strains and Culture Conditions

Strains ATCC 43504 and J99 of *H. pylori* were used in the study. Strains were incubated on microaerobic conditions (10% CO_2_, 5% O_2_, and 85% N_2_) for 3 days (ATCC 43504) or 2 days (J99) in Columbia agar containing 5% defibrinated horse blood and DENT. In all experiments, *H. pylori* cultures were examined under microscopy (Gram staining) in order to confirm its bacillary form. Also, urease and catalase activities were tested to assess the purity and viability of the cultures.

### Well Diffusion Assay

The well diffusion assay was used in the primary screening for the susceptibility of *H. pylori* ATCC 43504 and J99 strains to GTE and fractions. Bacterial suspensions, adjusted to yield approximately 6 × 10^8^ CFU/mL (McFarland N°2), were disseminated onto Müeller–Hinton agar plates containing 5% of defibrinated horse blood. The wells were made into the agar with a sterile Pasteur pipette and filled with 50 μL aliquots of total extract and fractions stock solutions on the inoculated agar surfaces. The plates were incubated for 5 days at 37°C under microaerobic conditions. Ellagic acid was used as natural product control and amoxicillin and metronidazole were used as antibiotic control. All tests were performed in triplicate, and the antibacterial activity observed was expressed as the mean of inhibition diameters (mm) produced by assayed samples.

### Minimal Inhibitory Concentration

MIC of *G. tinctoria* extracts against *H. pylori* ATCC 43504 and J99 was determined using a 96-microwell broth dilution method (Ngan et al., 2012; Biglar et al., 2014). Total extract and fractions of *G. tinctoria* plus standards ellagic acid and metronidazole were dissolved in 20 μL DMSO and diluted with water, and amoxicillin was dissolved and diluted with water. The solutions were sterilized with a sterile 0.22-μm filter. The inoculum was prepared in sterile physiologically serum to Mc Farland N°2 (∼6 × 10^8^ CFU/ml). An aliquot of 450 μL of the inoculum was taken and mixed with 42.300 μL brain heart infusion (BHI) broth and 2.250 μL of sterile fetal bovine serum (FBS). Columns 2 to 11 of the microwells were filled with 100 μL BHI broth. It was added to column 1 20 μL of the samples and 180 μL BHI broth. The column number one wells were mixed and took 100 μL to the next column, diluting serially twofold until column eleven. After that, 100 μL of the inoculum was added to columns from one to eleven. The negative control was 200 μL BHI broth and the positive control was 100 μL BHI broth and 100 μL of inoculum. The mixture was incubated and covered for 5 days at 37 °C under microaerobic conditions. The MIC concentration of extracts was defined as the lowest concentration inhibiting bacterial growth. Furthermore, 20 μL 0.01% resazurin (blue) was used, dissolved in PBS, and incubated at 37 °C under microaerobic conditions until it turned to resorufin (pink). If the well changed the color to pink, it meant that the bacteria remained viable.

### Minimal Bactericidal Concentration

For this assay, wells which showed no growth (MIC) were selected. Twenty microliters of each sample was taken (before resazurin addition), seeded on blood agar plates, and incubated for 72 h at 37 °C under microaerobic conditions in order to determine MBC, which was defined as the minimal concentration of sample required to kill 99.9% of the organisms in the medium after 72 h of incubation.

### Time-Kill Assay

For death kinetics assays, the bacterial inoculum was suspended in BHI broth supplemented with 5% FBS. Plant extracts were used at concentrations of 4 x MIC. The initial inoculum size was approximately 6 × 10^6^ CFU/mL. The samples were cultured at 37 °C under microaerobic conditions. Two aliquots for each time (0, 1, 2, 3, 4, 5, 8, 12, and 24 years 48 h) were obtained for the 43504 and J99 strains. Bacteria were counted using microdrop procedure (Miles et al., 1938) on Columbia agar supplemented with DENT (Flamm et al., 1996). Pearson's criteria were used to assess bacterial viability (Pearson et al., 1980). Bacterial counts were carried out in triplicate from two independent experiments. The second aliquot series (at 3 and 12 h) extracted from the experiment performed with the *H. pylori* 43504 strain were used for transmission electron microscopy (TEM).

### *Helicobacter pylori* Urease Extraction

To extract urease, *H. pylori* J99 strain was cultured in the conditions described above. The bacteria were harvested and let in sterile distilled water. The suspension was frozen and thaw seven times, using 60 s of sonication in each cycle of thawing. Following centrifugation (8.000 rpm for 10 min), the urease of the supernatant was precipitated with ammonium sulfate at 60%*.* The suspension was centrifuged and the precipitate was resuspended in 4 ml water and was desalted through a Sephadex G-25 column (Sigma). Fractions were collected and the ones which gave positive urease activity were pooled and mixed with an equal volume of glycerol and stored at −20 °C until use (Pastene et al., 2014).

### Urease Inhibition Assay

Evaluation of urease activity was carried out according to a previously published methodology (Tanaka et al., 2004). *H. pylori* and *Canavalia ensiformis* ureases (Jack Bean type IX urease; Sigma-Aldrich) were used, separately, in the assay mixture (25 μL, 4U) with 25 μL of different concentrations of GTE and its CPC fractions. Samples and urease were preincubated for 4 h at room temperature (for *H. pylori* urease, it was used at 37 °C) in a 96-well assay plate. After preincubation, 200 μL of 100 mM phosphate buffer at pH 6.8 containing 500 mM urea and 0.002% phenol red was added. The changes in absorbance at 570 nm were measured using Epoch Microplate Reader (BioTek).

### Measurement of Intracellular ATP Levels in *H. pylori*

Intracellular ATP was measured according to the protocol of Schweinitzer (Schweinitzer et al., 2008). Bacterial cells (from colonies in exponential growth phase) were resuspended in Brucella broth. Afterward, suspensions were incubated with GTE and CPC fractions for 1 h at 37 °C. The ATP content was performed with the Kit BacTiter-Glo reagent (Promega Inc., Madison, WI), incubating the samples for 5 min at 37 °C. Luminescence was measured with a Synergy HT multilector (BioTek Instruments, Vermont, USA). All experiments were performed at least three times on separate days in quadruplicate measurements (Pastene et al., 2009).

### Transmission Electron Microscopy

As mentioned above, the aliquots series obtained from the time-kill curve with the *H. pylori* 43504 strain were used for TEM (Romero et al., 2019). Bacteria were collected by centrifugation, fixed in 2.5% glutaraldehyde, and kept at 4°C for one week until the moment they were processed. The samples were processed and analyzed in the Electronic Microscopy Center of the University of Concepción. The samples were washed in cacodylate buffer (pH 7.4) at 4°C for 12 h and then postfixed in 1% osmium tetroxide in the same buffer for 2 h. The specimens were then dehydrated through a graded series of acetone (30°, 50°, 70°, 90°, and 100°), exchanged through propylene oxide, and embedded in a mixture of epoxy resin (Durcupan ACM, Fluka, Switzerland). Sections of about 100 nm were obtained by a microtome with a glass knife and were stained with 1% uranyl acetate for 10 min, followed by a lead staining reagent. The sections were examined with a transmission electron microscope (JEOL-JEM 1200 EX II, Jeol Technics Ltd., Tokyo, Japan).

### Statistical Analysis

Data were analyzed using the GraphPad Prism 5 statistical software, by analysis of variance (ANOVA) and Tukey’s multiple comparison test.

## Results and Discussion

### Metabolomic Analyses of *G. tinctoria*

**FIGURE 2:**
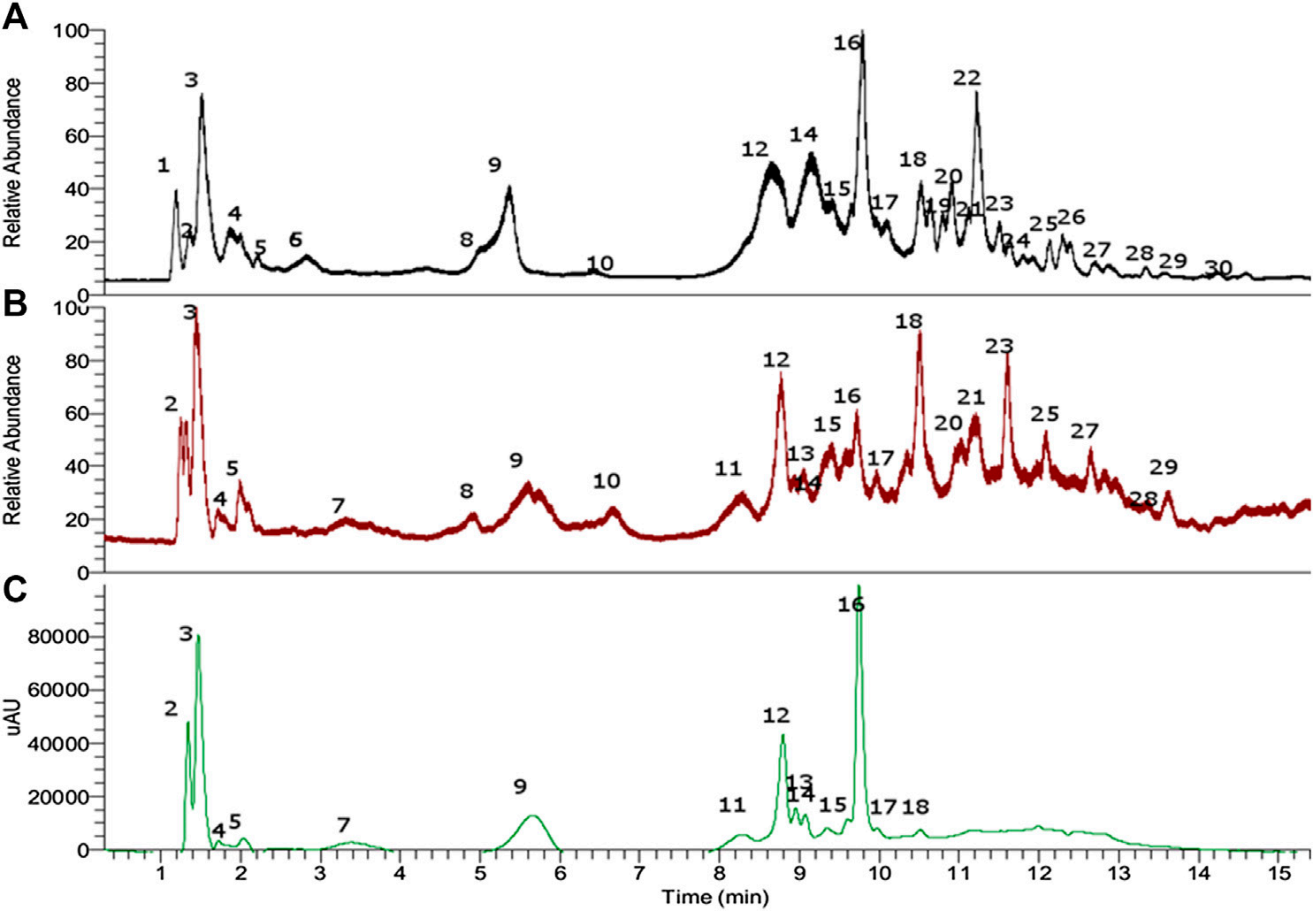
UHPLC chromatogram of petioles extract of *Gunnera tinctoria*. **(A)** TIC negative mode, **(B)** TIC positive mode, and **(C)** PDA chromatogram (520 nm).

Electrospray orbitrap (EI-OT) became a very rapid and versatile tool for the fast identification of fruit and edible plant materials (Simirgiotis et al., 2016b; Simirgiotis et al., 2016c). In this study, a UHPLC fingerprint was generated using EI-OT-HR-MS (Figure 2), allowing for the determination of several metabolites in the petioles of the Mapuche medicinal food species *G. tinctoria* (Table 1). In addition, in order to obtain a polyphenolic-enriched extract from the edible petioles of *G. tinctoria*, the following procedure was conducted. The petioles (1.5 kg fresh) were extracted with hot water first and then with 95% ethanol. After removal of ethanol under vacuum, sugars and other nonphenolic substances of *G. tinctoria* aqueous extract were cleaned on a macroporous resin (Sepabeads SP-850). This procedure enabled obtaining 27 g of dried extract (GTE), which is equivalent to a yield of 1.8% on a wet basis. Considering that water content in fresh petioles was 78.02 ± 0.1%, the GTE yield was 8.19% on the dried basis. This extract was analyzed using LC Orbitrap mass spectrometry. Electrospray Quadrupole Orbitrap^®^ became a versatile and very rapid tool for the characterization of phenolics in plants including toxins and pigments (Simirgiotis et al., 2016a; Simirgiotis et al., 2016b; Simirgiotis et al., 2016c). This state-of-the-art technology was used to determine the metabolomic profiles of *G. tinctoria* and to set up chemical fingerprints for chemotaxonomy and identification of the plant material since this species is largely sold as food in the street markets in Chile (Zamorano et al., 2017). Figure 2 shows the UHPLC chromatograms of *G. tinctoria* extract as follows: 1) total ion current (TIC) chromatogram in ESI negative mode; 2) total ion current chromatograms in ESI positive mode; and 3) UV chromatogram at 520 nm. Below is the detailed explanation of the rapid metabolome analysis of the aforementioned unstudied *Mapuche* medicinal food using this high-resolution accurate mass spectrometry (HRAM) technique for the first time. The details of full MS spectra and structures of peaks 1, 7-10, 18, 23-26, and 28 are presented in Supplementary Figure S1 (Supplementary Files)

**TABLE 1:** Full metabolome analysis: UV maxima and high-resolution Q-Orbitrap MS data and formulas for the metabolites identified in Nalca (*G. tinctoria*) extract.

Peak #	t_R_ (min)	λmax (nm)	Elemental composition [M-H] ^−^	Theoretical mass (*m/z*)	Measured mass (*m/z*)	Accuracy (δppm)	Other HR ions (*m/z*)	Tentative identification	Ref
1	1.20	-	C_6_H_7_O_7_^−^	191.01973	191.01930	2.14		Citric acid	Zamorano et al. (2017)
2	1.35	185	C_7_H_5_O_5-_	169.01315	169.01379	3.81		Gallic acid^a^	Brito et al. (2014); Shanmuganathan and Angayarkanni (2018)
3	1.45	277–514	C_27_H_31_O_16_^+^	611.16121	611.17700	25.8	287.0552 (cyanidin)	Cyanidin 3-*O*-(2”-*O*-glucosyl) glucoside	Tsuda et al. (1994); Miyazawa et al. (1999); Delazar et al. (2010); Avello Lorca et al. (2016)
4	1.75	277–514	C_27_H_31_O_16_^+^	611.16121	611.16189	1.12	287.0546 (cyanidin)	Cyanidin 3-*O*-(2”-*O*-glucosyl) galactoside	Tsuda et al. (1994); Miyazawa et al. (1999); Delazar et al. (2010); Avello Lorca et al. (2016)
5	2.05	316–512	C_27_H_31_O_16_^+^	611.16121	611.16164	0.70	287.0546 (cyanidin)	Cyanidin 3,5-di-*O*-glucoside	Tsuda et al., (1994); Miyazawa et al. (1999); Delazar et al. (2010); Avello Lorca et al. (2016)
6	2.47	325	C_16_H_19_O_9_^−^	355.10346	355.07846	−70		Feruloyl glucoside	Jeong et al. (2011)
7	3.54	288–512	C_27_H_31_O_16_^+^	611.16121	611.16219	1.60	287.0546 (cyanidin)	Cyanidin 3,7-di-*O*-glucoside	Ruiz et al. (2010)
8	4.78	325	C_16_H_19_O_9_^−^	355.10346	355.09356	−25	Feruloyl glucoside	Feruloyl galactoside	Jeong et al. (2011)
9	5.63	277–514	C_28_H_33_O_16_^+^	625.17686	625.17643	−0.68	321.07116 (peonidin)	Peonidin 3-*O*-(2”-*O*-glucosyl) galactoside	Passamonti et al. (2002); Ruiz et al. (2010)
10	9.78	310	C_15_H_17_O_8_^−^	325.0929	325.09649	11.04		p-Coumaroyl glucoside	Bystrom et al., (2008)
11	8.25	277–514	C_28_H_33_O_16_^+^	625.17686	625.17678	−0.12	301.07112 (peonidin)	Peonidin 3-*O*-(2”-*O*-galactosyl) galactoside	Passamonti et al. (2002)
12	8.78	277–514	C_28_H_33_O_16_^+^	625.17686	625.19348	26.58	301.07112 (peonidin)	Peonidin 3,5-di-*O*-glucoside	Passamonti et al. (2002); Ruiz et al. (2010)
13	8.87	277–514	C_29_H_35_O_16_^+^	639.19251	639.19223	−0.23	301.07112 (peonidin)	Peonidin 4’ *O-*methyl*-*3-*O*-(2”-*O*-glucosyl) galactoside	Passamonti et al. (2002); Ruiz et al. (2010)
14	9.03	277–514	C_29_H_35_O_16_^+^	653.20816	653.20801	−0.22	301.07112 (peonidin)	Peonidin 7, 4’ *di-O-*methyl*-*3-*O*-(2”-*O*-glucosyl) galactoside	
15	9.40	277–514	C_22_H_23_O_11_^+^	463.12404	463.12403	3.9	301.07112 (peonidin)	Peonidin-3-*O*-glucoside	Passamonti et al. (2002); Ruiz et al. (2010)
16	6.57	325	C_20_H_17_O_14_^−^	483.07803	483.07800	−0.06		Digalloylglucose	Mena et al. (2012); Shanmuganathan and Angayarkanni (2018)
17	9.95	277–512	C_21_H_21_O_11_^+^	449.10839	449.11520	15.16		Chrysanthemin (cyanidin-3-*O-*glucoside)^a^	Ruiz et al. (2010); Ramirez et al. (2015)
18	10.45	277–512	C_15_H_11_O_6_^+^	287.05556	287.05548	−1.60		Cyanidin^a^	Ruiz et al. (2010)
19	10.32	256–353	C_27_H_29_O_16_^−^	609.14611	609.14537	−1.21	301.03512 (quercetin)	Rutin^a^	Simirgiotis et al. (2013)
20	10.63	254–354		447.09329	447.09316	3.55		Quercetin-3-*O*-rhamnoside	Huang et al. (2018)
21	11.02	256–353	C_22_H_21_O_12_^−^	477.10385	477.10273	−2.17	315.05093 (isorhamnetin) 300.02771	Isorhamnetin-3-*O*-glucoside	Brito et al. (2014)
22	11.43	254–365	C_27_H_29_O_15_^−^	593.15070	593.15010	1.01	285.04013 (kaempferol)	Kaempferol-3-*O*-rutinose	Simirgiotis et al. (2015)
23	11.55	325	C_41_H_27_O_26_^−^	935.07960	935.07501	−4.90	300.99906	Potentillin	Hager et al. (2008); Piwowarski et al. (2016)
25	12.23	325	C_41_H_29_O_26_^−^	937.09525	937.08966	−5.96	300.99908	Tellimagrandin	Regueiro et al. (2014); Takeuchi et al. (2017)
24	11.66	325	C_20_H_17_O_14_^−^	481.06238	481.06259	0.43	300.99856	HHDP-glucose	Mena et al. (2012)
26	12.43	281	C_14_H_5_O_8_^−^	300.99899	300.99855	−1.46		Ellagic acid^a^	Lee et al. (2005)
27	12.83	324	C_9_H_7_O_4_^−^	179.03443	179.03441	−0.11		Caffeic acid^a^	Simirgiotis et al. (2013)
28	13.22	325	C_41_H_29_O_27_^−^	953.09017	953.08514	−5.27	300.99856	Chebulagic acid	Regueiro et al. (2014); Shanmuganathan and Angayarkanni (2018)
29	13.53	255–275	C_11_H_11_O_6_	239.0560	239.05501	4.12		Unknown	
30	14.20	254–354	C_15_H_9_O_7_^−^	301.03538	301.03545	0.23	179.01254	Quercetin^a^	Simirgiotis et al. (2013)

^a^ Identification based on HR-MS data and comparison with certified standard.

### Simple Organic Acids

Peak 1 with ion *m/z* 191.01933 was determined as citric acid (Brito et al., 2014); this compound was already reported and quantified in *G. tinctoria* (Nalca) (Zamorano et al., 2017).

### Anthocyanins

Peaks 3 to 5 and 7, all of them showing a molecular cation around m/z: 611, were identified as isomers of a diglycosylated cyanidin structure, particularly cyanidin 3-*O*-(2”-*O*-glucosyl) glucoside, cyanidin 3-*O*-(2”-*O*-glucosyl) galactoside (Tsuda et al., 1994; Miyazawa et al., 1999; Delazar et al., 2010; Ruiz et al., 2010), cyanidin 3,5-di-*O*-glucoside, and cyanidin 3,7-di-*O*-glucoside, respectively (Table 1). In a similar manner, several compounds were identified as peonidin glycoside derivatives, such as peonidin-3-*O*-glucoside (Miyazawa et al., 1999), peak 15 (λ_max_: 277–514). Peaks 9, 11, and 12, all glycosylated isomers of peonidin with a molecular cation at around 625 Da, were identified as peonidin 3-*O*-(2”-*O*-glucosyl) galactoside, peonidin 3-*O*-(2”-*O*-galactosyl) galactoside, and peonidin 3,5-di-*O*-glucoside (Passamonti et al., 2002), respectively. Peak 13 with a [M]^+^ cation at *m/z*: 639.19243 was identified as peonidin 4’ *O-*methyl*-*3-*O*-(2”-*O*-glucosyl) galactoside (C_29_H_35_O_16_), while peak 14 was identified as peonidin 7, 4’ *di-O-*methyl*-*3-*O*-(2”-*O*-glucosyl) galactoside (C_29_H_35_O_16_). Peak 17 was identified as cyanidin-3-*O-*glucoside (Ramirez et al., 2015) and peak 18 as cyanidin by spiking experiments with authentic standards.

### Hydrolyzable Tannins

Peak 24 with a [M-H]^-^ ion at *m/z*: 481.06259 was identified as HHDP-glucose (C_20_H_17_O_14_^−^) and peak 16 as the related derivative digalloylglucose (C_20_H_17_O_14_^−^), while peak 23 with a [M-H]^-^ ion at *m/z*: 935.07501 was identified as potentillin (C_41_H_27_O_26_^−^), peak 28 as chebulagic acid (C_41_H_29_O_27_) (Shanmuganathan and Angayarkanni, 2018), and peak 25 as tellimagrandin (C_41_H_29_O_26_^−^).

### Flavonoids

Peaks 19–22 and 30 were identified as flavonol derivatives since the shape of the UV spectra was similar to that reported elsewhere (Mabry et al., 1970; Sun et al., 2007; Simirgiotis et al., 2009). Peak 19 with a [M-H]^-^ ion at *m/z*: 609.14537 was identified as rutin (C_27_H_29_O_16_); peak 20 showing a pseudomolecular ion at *m/z*: 447.09316 was identified as the quercetin 3-*O*-rhamnoside (quercitrin). This compound is common in Myrtaceae plants and was identified by our group in *Ugni molinae* (Rubilar et al., 2006; Simirgiotis et al., 2013). Peak 21 (*m/z*: 477.10385, [M-H]^−^) was consistent with the molecular formula C_22_H_22_O_12_, corresponding to isorhamnetin-3-*O*-glucoside (Gutzeit et al., 2007). Peak 22 with a parent ion at *m/z*: 593.15010 was identified as kaempferol-3-*O*-rutinose (C_27_H_29_O_15_) (Sánchez-Montoya et al., 2017) and peak 22 as quercetin.

### Phenolic Acids

Peak 2 was identified as gallic acid (Brito et al., 2014; Shanmuganathan and Angayarkanni, 2018), peak 6 as feruloyl glucoside (C_16_H_19_O_9_^−^) (Lee et al., 2005), and peak 8 as its isomer feruloyl galactoside, while peak 10 was identified as the related derivative p-coumaroyl glucoside (C_15_H_17_O_8_^−^), (Lee et al., 2005), respectively. Peaks 26 and 27 with pseudomolecular ions at *m/z*: 300.99899 and 179.03441 were identified as ellagic (Lee et al., 2005) and caffeic acids, respectively (C_11_H_11_O_6_ and C_9_H_7_O_4_). Only caffeic acid was already reported in Nalca leaves (Zamorano et al., 2017).

### Unknown

Peak 29 was an unidentified compound (C_11_H_11_O_6_).

### Bioactivity-guided fractionation of the total extract and anti-*H. pylori* activity

**FIGURE 3:**
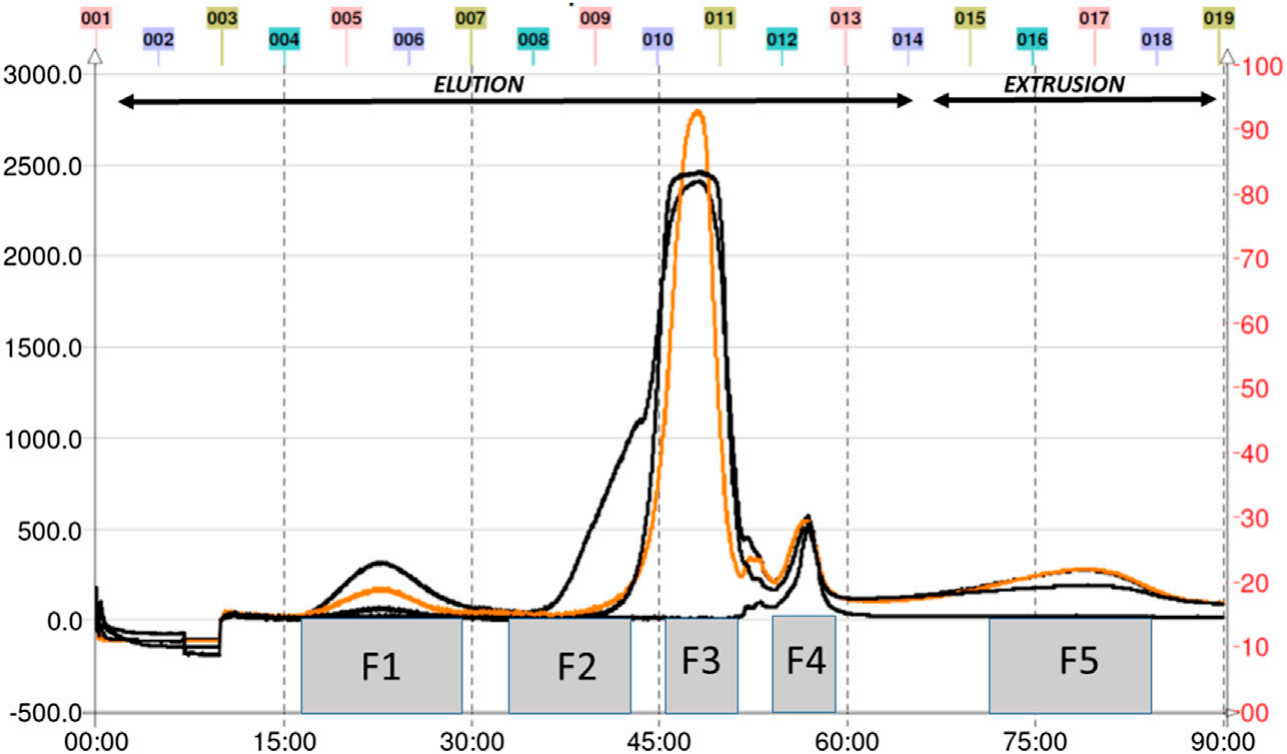
CPC chromatogram of petioles extract of *Gunnera tinctoria*.

**FIGURE 4:**
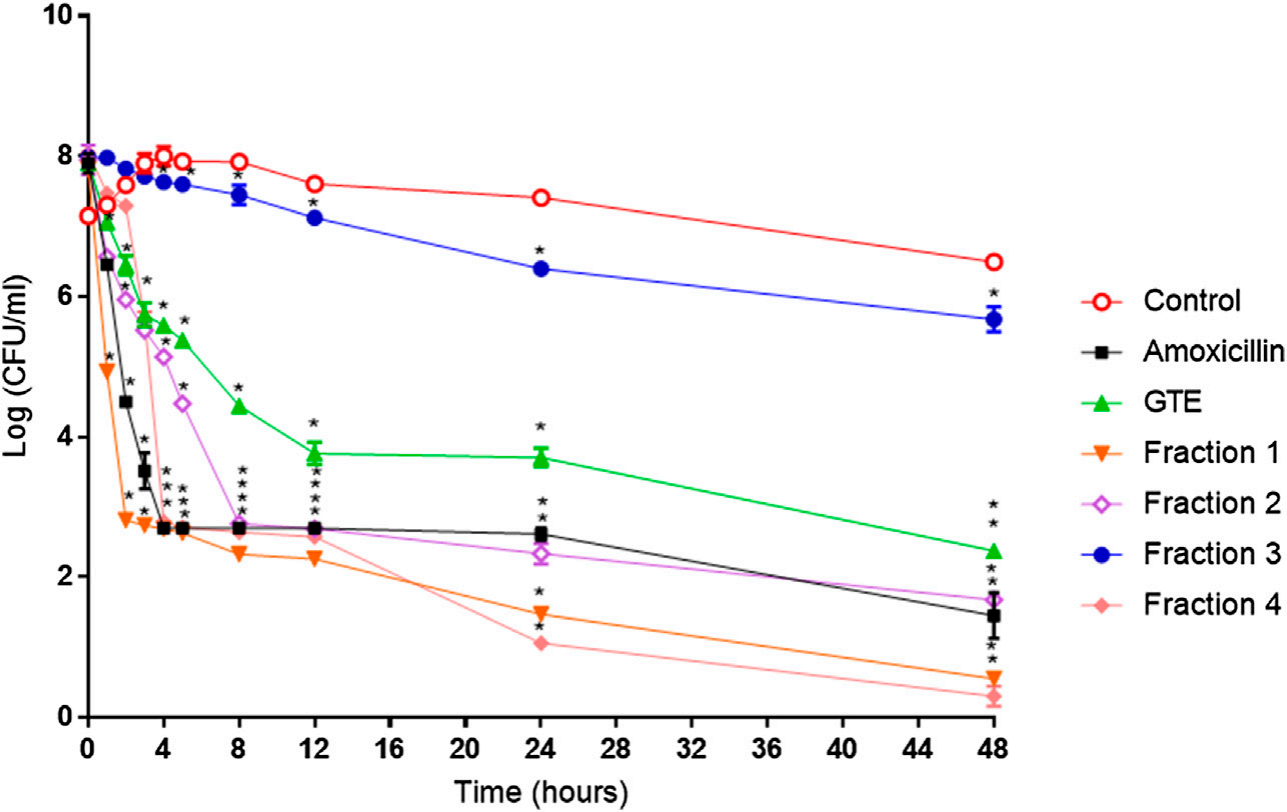
*Helicobacter pylori* ATCC 43504 death kinetics with *G. tinctoria* extracts and its fractions obtained by Centrifugal Partition Chromatography (CPC). (○) Control without extracts, (■) amoxicillin (0.25 μg/ml), (▲) *Gunnera tinctoria* extract (GTE, 256 μg/ml), (▼) fraction 1 (512 μg/ml), (◊) fraction 2 (512 μg/ml), (●) fraction 3 (512 mg/ml), and (♦) fraction 4 (1,024 μg/ml).*Time in which significant growth differences are observed (*p* ≤ 0.05) as determined by variance analysis followed by Tukey’s test. All points are the average of two independent experiments with three replicas each.

**FIGURE 5:**
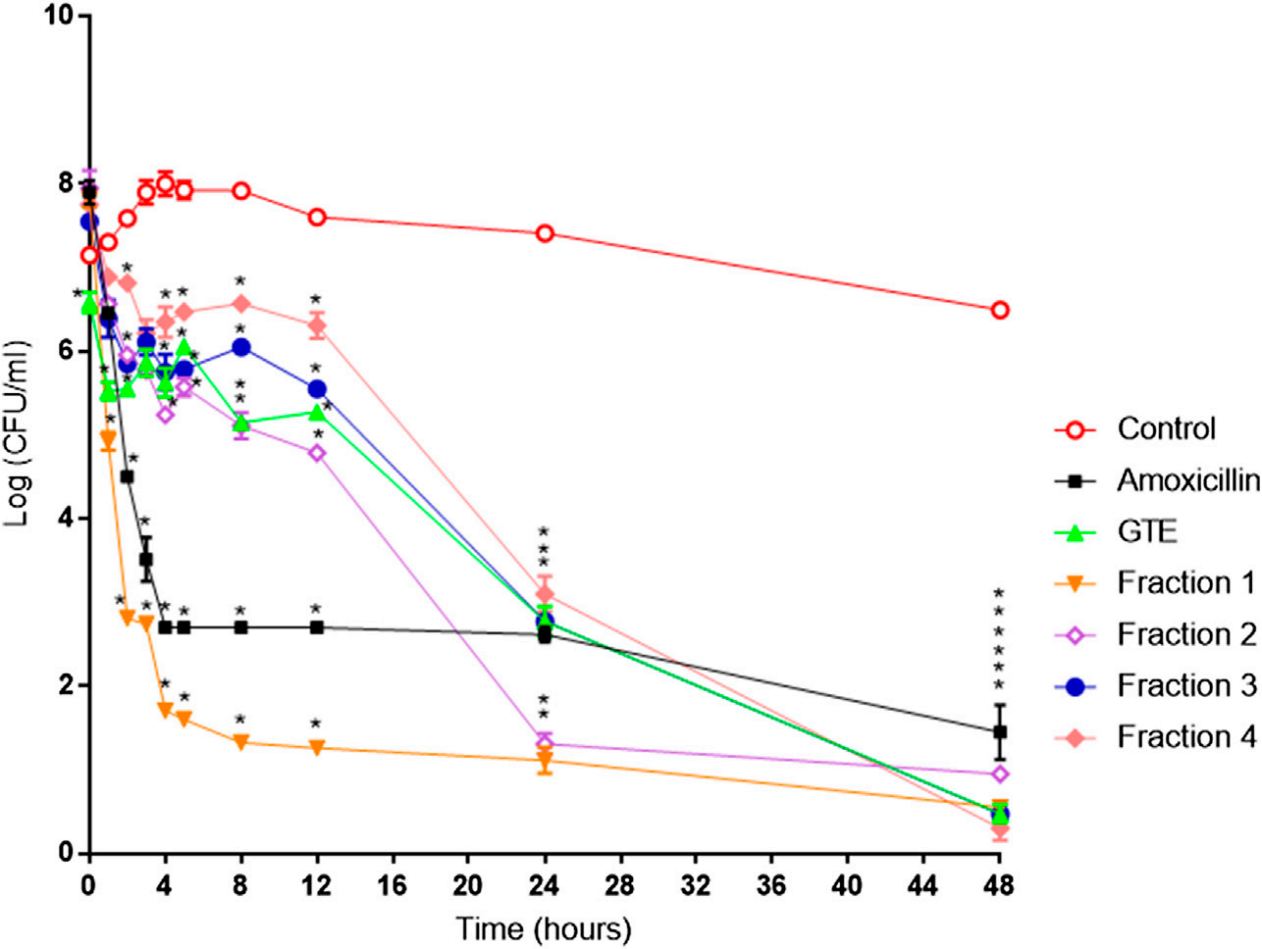
*Helicobacter pylori* ATCC J99 death kinetics with *G. tinctoria* extracts and its fractions obtained by Centrifugal Partition Chromatography (CPC). (○) Control without extracts, (■) amoxicillin (0.25 μg/ml), (▲) *Gunnera tinctoria* extract (GTE, 256 μg/ml), (▼) fraction 1 (512 μg/ml), (◊) fraction 2 (512 μg/ml), (●) fraction 3 (512 mg/ml), and (♦) fraction 4 (1,024 μg/ml).*Time in which significant growth differences are observed (*p* ≤ 0.05) as determined by variance analysis followed by Tukey’s test. All points are the average of two independent experiments with three replicas each.

**FIGURE 6:**
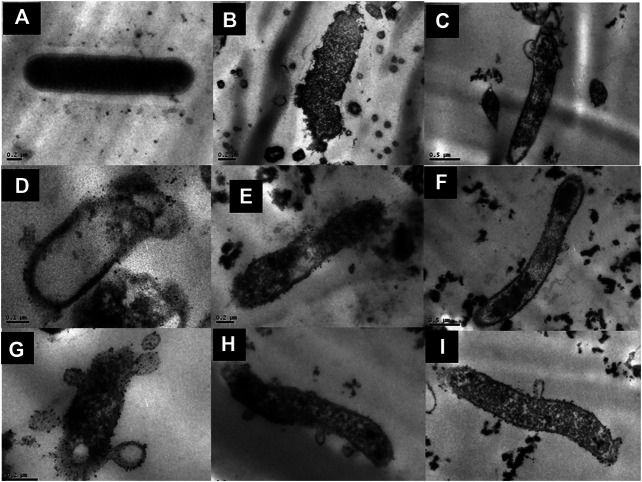
Structural effect of *G. tinctoria* extracts and its fractions obtained by Centrifugal Partition Chromatography (CPC) upon *Helicobacter pylori* ATCC 43504. Bacterial cells were exposed for 3 or 12 h with the extracts and fractions and analyzed by transmission electron microscopy (see *Material and Methods*). **(A)** Rod-shaped *Helicobacter pylori* without extracts, **(B)** amoxicillin treatment (0.25 μg/ml), **(C)** *G. tinctoria* extract GTE (256 μg/ml), **(D)** F1 fraction (512 μg/ml), **(E)** F2 fraction (512 μg/ml), **(F)** F3 fraction (512 μg/ml), **(G)** F4 fraction (1,024 μg/ml), **(H)** *H. pylori* incubated for 3 h with F1 fraction, and **(I)** *H. pylori* incubated for 3 h with F4 fraction at different magnification. The values of bars used were A, B, E, G, H, and I: 0.2 μm; C and F: 0.5 μm; D: 0.1 μm.

The total aqueous extract of *G. tinctoria* petioles (GTE) was fractionated using CPC in order to identify compounds with anti-*H. pylori* activity. This technique allowed the fractionation of highly water-soluble target compounds according to its K values (Figure 3). Therefore, in ascending mode, the order of elution was related to polarity being F1 < F2 < F3 < F4 < F5 (highly polar compounds in the extrusion step). After HPLC-MS analysis, we tentatively identify in F2 three phenolic compounds: digalloylhexose, pentagalloylhexose, and potentillin (Table 2). Interestingly, this fraction gave the best results in the well diffusion assay (Tables 3 and 4) and also in the other bioassays. Moreover, in fraction F1, we identified mainly phenolic acid glycosides such as p-coumaroyl hexose and two ferulic acid hexoside, whereas in F3, the main compound was tellimagrandin. As expected, only polymeric material was obtained after the CPC extrusion step (Fraction 5). Therefore, the identification of compounds in F5 was not possible using UHPLC-Q-Orbitrap-HR-MS in reverse-phase column. Several phenolic compounds obtained by CPC are the same as that tentatively identified in Table 1. Additionally, several anthocyanins were identified in GTE, but their contribution to the total phenolic profile was quite low in comparison with phenolic acid glycosides and the derivatives of gallic and ellagic acids. In the study of Zhang and colleagues (Zhang et al., 2013), it was determinate that the compound 1,2,3,6-tetra-O-galloyl-β-D-glucose extracted from *Geranium wilfordii* had MICs between 2 and 8 μg/ml against *H. pylori* 43504 strain and upon five other clinical isolate strains. In the well diffusion assay (Table 3), it was found that the inhibition halos for the 43504 strain obtained with GTE and F2 were very similar. Thus, this data suggested that the structural similarity of these molecules with other galloylglucose derivatives could be responsible for their activity. Considering that F2 was the most abundant in GTE, it is clear that its antibacterial activity could be mainly associated with the compounds of this fraction. The same results were observed when GTE and F2 were tested against J99 strain (Table 4). The fact that J99 strain was more affected than 43504 strain could be explained because the first one is a fast-growing strain. In the MIC assay, according to the Wang classification (Wang, 2014), the GTE and all its fractions would have a high-moderate activity against the strain J99, being the MICs range between 10 and 100 μg/ml (Table 4). Using Wang's criterion, MIC range between 100 and 1,000 μg/ml (Table 3) suggests that GTE has a weak to moderate activity against 43504 strain. Also, this result is interesting since *H. pylori* 43504 strain is resistant to metronidazole, which is an antibiotic widely used as part of the recommended eradication therapy. Regarding this latter, Wang and coworkers analyzed 34 studies in which the anti-*H. pylori* effect of 80 plants was reported. In this study, just one plant shows a high activity (2.9%) against *H. pylori* and four plants show a high to moderate activity (11.8%). Most of them displayed low activity, being 17 studies (50%) with a low to moderate activity and 11 (32.4%) with low activity (Wang, 2014). In an Indian study, GutGard^®^, a commercial flavonoid-rich extract of *Glycyrrhiza glabra*, showed a similar *in vitro* activity over a collection and clinical isolates of *H. pylori* strains, obtaining values between 32 and 64 μg/ml (Asha et al., 2013). In our work, MIC values for GTE and the chromatographic fractions against *H. pylori* J99 strain were lower than those values found for *H. pylori* 43504 strain. We think that this result is linked with the higher growth rate of the J99 strain because in the logarithmic phase of growth the bacteria are more sensitive to antimicrobial molecules (Millar and Pike, 1992). In contrast, the slow growth rate of *H. pylori* 43504 could explain, at least in part, the lesser sensitivity of this strain to *G. tinctoria* extracts. This statement is in line with previous reports where the slow growth rate of *H. pylori* is considered an important mechanism of resistance because in acid environment the permeability of bacterial cell membrane is altered, increasing MIC values for several antibiotics (Kusters and Kuipers, 2001; Mégraud and Lamouliatte, 2003). The 43504 strain showed metronidazole resistance, with a MIC 32 μg/ml, which is similar to the values reported previously (Ngan et al., 2012). As MICs and MBCs had the same values, the GTE and *G. tinctoria* fractions behave as a bactericide *in vitro*. In the time-kill curve, F1 acts more rapidly than amoxicillin for the two tested strains, having a lethal effect at 2 h and 3 h for 43504 strain (Figure 4) and for J99 strain (Figure 5), respectively. Considering that the maximum gastric emptying time is around 4 h, F1 could have enough time to act against *H. pylori*. The positive control in the time-kill curve for both strains shows a mild decrement in its growth at 24 and 48 h (Figures 4, 5). This effect could be associated with the initial bacterial inoculum (5.7 × 10^6^ CFU/ml). Additionally, the environment and nutrients can promote bacterial multiplication, generating toxic metabolic wastes. Altogether, these conditions result in a diminution of nutrients and a reduction in the bacterial count after 24 h. The same effect can be seen for the F3 fraction, which at the 48 h did not display a lethal effect upon the bacteria, making the same behavior observed for the positive control. For the *H. pylori* J99 strain, F3 displays antibacterial activity, although the count decreased three logarithms (bacterial death according to Pearson criteria) just after 24 h. Urease is one of the main virulence factors of *H. pylori*, which is essential to its survival in the acidic environment of the stomach. The results (IC_50_) of urease inhibition assay are presented in Table 4 for GTE and all *G. tinctoria* fractions. These values suggest that *G. tinctoria* could prevent the early steps of colonization of this pathogen in the human stomach. In the study of Biglar and coworkers (Biglar et al., 2014), the capacity of 20 Iranian plants to inhibit Jack Bean urease was evaluated, finding that just five of these plants had IC_50_ lower than 100 μg/ml. For this last study, the IC_50_ values ranged between 786.71 and 48.54 μg/ml. In the present work, besides the evaluation of the inhibition of Jack Bean urease, we assessed the inhibitory effect upon *H. pylori* urease. So, for the vegetal urease, IC_50_ values were between 35.6 μg/ml (F4) and 19.4 μg/ml (F2), whereas for the bacterial urease, IC_50_ values ranged between 52.4 μg/ml (GTE) and 13.5 μg/ml (F3) (Table 4). Interestingly, although F3 displayed a poor activity in the time-kill curves for *H. pylori* 43504 strain, it has a high inhibitory effect upon its urease. These results suggest that F3 fraction only affects *H. pylori* viability via urease inhibition. In fact, as is seen in Figure 6 (frame F), at 12 h of incubation, this fraction did not affect *H. pylori* morphology in comparison with F1, F2, and F4 and behaves in a similar way to the control experiments without extracts. In the present work, TEM images (Figure 6A-I) suggest that GTE and F1, F2, and F4 fractions obtained by CPC induce disruption and separation of the cellular wall and outer membrane detachment on *H. pylori*. Moreover, at 3 h, F1 induces early blebs formation in *H. pylori* 43504 strain (Figure 6H), and at 12 h, all the bacterial morphology is altered, which is evidenced by the massive presence of empty envelops and leaking of cytoplasmic contents into the intermembrane space (Figure 6D). Although fewer in number, early blebs also are observed in *H. pylori* incubated with F4 fraction for 3 h (Figure 6I). On the other hand, after 12 h of incubation with GTE, F2, and F4, blebbing of the outer membrane, lysis, and secretory granule loss are evident in the presence of such fractions (Figures 6C,E,G **)**. Regarding these TEM results, Horii and coworkers (Horii et al., 2002) found that β-lactamase inhibitors like clavulanate and sulbactam produce changes in the cellular morphology, the disintegration of the cellular wall, and lysis of *H. pylori*. All these morphological alterations lead to explosive cell lysis of *H. pylori* induced by the *G. tinctoria* phenolic compounds. Similar results were obtained using urushiol from *Rhus vernicifera Stokes* (Suk et al., 2011), with the resin of the tree *Pistacia lentiscus* (Marone et al., 2001) and by our group with Propolis extracts (Romero et al., 2019). In order to understand how GTE and its CPC fractions decrease bacterial viability, an additional assay was performed using *H. pylori* ATCC 43504 strain through the measurement of intracellular ATP. Analysis of ATP is a suitable indicator of bacterial viability since coccoid forms of *H. pylori* have 100-fold to 2000-fold lower ATP levels than the spiral forms (Krzyżek and Grande, 2020). In Supplementary Figure S2 (Supplementary files), GTE, F1, F2, and F4 decreased *H. pylori* intracellular ATP levels in a concentration-dependent manner. Interestingly, it was found that after 1 h of incubation GTE extracts largely reduce bacterial intracellular ATP levels around 60–70%. In the case of F3, the reduction of ATP levels only falls near 30%. These results are consistent with those observed in the death kinetics curves and TEM experiments. In fact, after a 1-h incubation of *H. pylori* with 500 μg/ml of F1 and F4, the decrease in the ATP levels is close to 60%. This observation is in line with the visible induction of vesiculation in samples treated with F1 and F4 (Figures 6, frames H and I) for 3 h. In a similar approach, Nagata and coworkers (Nagata et al., 2001) reported the concentration-dependent effect of lansoprazole on ATP levels on *H. pylori*. However, in this study even when respiratory activity was completely suppressed from lansoprazole concentrations of 100 μg/ml, it was not possible to decrease ATP levels beyond 20%. The authors concluded that there is an ATP pool that is insensitive to lansoprazole and that it is probably not associated with the respiratory activity of *H. pylori*. Moreover, it has been reported that the treatment with lansoprazole induced cell elongation and alterations to the cell surface (Nakao et al., 1995). Another explanation regarding the effect of polyphenols upon morphological changes in *H. pylori* cell wall underlaid the interaction with penicillin-binding protein subunits (PBP). This mechanism has been recently described for silybin (Bittencourt et al., 2020). However, other mechanisms could not be ruled out. For instance, blebbing also could be caused for drugs like miconazole without the induction of coccoid forms of *H. pylori* (Von Recklinghausen et al. 2020). In a study of Annuk and coworkers (Annuk et al., 1999), they tested a series of plant extracts and found that tannic acid contained in bearberry alters the cell surface hydrophobicity (CSH) of *H. pylori*. On the other hand, disruption of glycocalyx-cell wall is a common process associated with the action mechanism of some polycationic agents and antibiotics like gentamicin and polymyxin (Nicas and Hancock, 1980). Indeed, bismuth salts used as a second-line treatment of *H. pylori* infection (Malfertheiner et al., 2017) interact with the glycocalyx external to the outer membrane promoting cell wall distortion and blebbing (Stratton et al., 1999), among other mechanisms not yet fully understood (Ge et al., 2012). Furthermore, for a drug to have an effect on this pathogen, it must not only dissolve and be stable in the acidic environment of the stomach but also be able to penetrate the thick mucus layer in which *H. pylori* is embedded. In this sense, any agent that contributes to disintegrating these barriers could act cooperatively with the pharmacological eradication therapy (Roesler et al., 2012). As can be seen in Tables 1, 2, Nalca predominantly possesses phenolic compounds of the tannin group. These substances *in vivo* could not only act directly against *H. pylori* (Funatogawa et al., 2004), through some of the mechanisms described above but also have beneficial effects on the gastric mucosa. For example, they can precipitate proteins at the site of a peptic ulcer, forming a protective barrier against proteolytic enzymes and the absorption of toxic substances. Furthermore, early works had demonstrated the inhibitory activity of ellagic and tannic acids upon gastric H^+^ and K^+^-ATPase, enabling a decrease in HCl secretion (Murakami et al., 1991; de Jesus et al., 2012). In the same way, Khennouf et al. reported that pedunculagin, phillyraeoidin A, castalagin, and acutissimin B isolated from different Quercus species showed a gastroprotective effect on ethanol-induced gastric ulcers in mice (Khennouf et al., 2003). These last compounds are structurally closely related to the hydrolyzable tannins reported for *G. tinctoria* in the present study. A recent work (Zamorano et al., 2017) showed that *G. tinctoria* leaf extracts display high antioxidant activity and inhibit the growth of *Cladophialophora*’s mold and the yeast *Cryptococcus laurentii*. In line with these results, Velásquez et al. (2020)( reported that ethanol extracts of *G. tinctoria* inhibit the growth of *Pseudomonas aeruginosa*, *Staphylococcus aureus*, and *Escherichia coli* with MIC values of 4.7 mg/ml, that is, 150 times greater than MIC values observed by us for GTE upon *H. pylori*. To the best of our knowledge, this is the first study of the anti-*H. pylori* activity of *G. tinctoria*. Finally, it is worth noting that the World Health Organization (WHO), in its “global priority list of antibiotic-resistant bacteria to guide research, discovery, and development of new antibiotics”, places *H. pylori* in priority 2 (high). This is due to the worrying increase in its resistance against clarithromycin. In this sense, the report raises the urgent need to seek new treatments and strategies against this bacterium (Tacconelli et al., 2018).

**TABLE 2:** Major metabolites identified in fractions of Nalca (*G. tinctoria*) obtained by Centrifugal Partition Chromatography.

CPC fraction	Peak	t_R_ (min)	λmax (nm)	Elemental composition [M-H] ^−^	Theoretical mass (*m/z*)	Measured mass (*m/z*)	Accuracy (δ ppm)	Ions MS^2^ (*m/z*)	Tentative identification	Ref
**F1**	6		274	C_16_H_19_O_9_^−^	355.10346	355.07846	−70	265.4; 145.2; 163.2	Ferulic acid glucoside	Jeong et al. (2011)
	8		283; 310 (sh)	C_16_H_19_O_9_^−^	355.10346	355.09356	−25	295.3; 175.2; 191.3; 235.3; 149.2	Ferulic acid galactoside	Jeong et al. (2011)
	10		280; 325 (sh)	C_15_H_17_O_8_^−^	325.0929	325.09649	11.04	284.3; 229.4	p-Coumaroyl hexose	Bystrom et al. (2008)
	26		282; 326 (sh)	C_14_H_5_O_8_^−^	300.99899	300.99855	−1.46	295.3; 193.2; 175.2; 191.3; 160.2	Ellagic acid	Lee et al. (2005)
	28		253	C_41_H_29_O_27_^−^	953.09017	953.08514	−5.27	301.3; 275.3 301.3	Chebulagic acid	Regueiro et al. (2014); Shanmuganathan and Angayarkanni (2018)
**F2**	16	6.9	273	C_20_H_17_O_14_^−^	483.07803	483.07800	483.7	313.4; 331.4; 169.2	Digalloylhexose	Lee et al. (2005)
	23	18.6	275	C_41_H_27_O_26_^−^	935.07960	935.07501	−4.90	301.3; 249.3; 275.4; 313.3; 169.2	Potentillin	Lee et al. (2005)
	26	21.1	367	C_14_H_5_O_8_^−^	300.99899	300.99855	301.6	284.2; 229.3; 185.2	Ellagic acid	Abu-Reidah et al. (2015)
**F3**	25	19.8	276	C_41_H_29_O_26_^−^	937.09525	937.08966	−5.96	301.5; 767.6; 465.5; 749.8; 451.6	Tellimagrandin	Regueiro et al. (2014); Shanmuganathan and Angayarkanni (2018)
**F4**	All other anthocyanins and flavonoids were detected in this fraction (see Table 1)									
**F5**	Polymeric fraction with no activity. It was not possible to identify molecules in this fraction									

**TABLE 3:** Well diffusion assay, MIC, and MBC for *H. pylori* strain 43504.

Extract/CPC fraction	*H*. *pylori* strain 43504						
	Concentration (µg/ml)					MIC (µg/ml)	MBC (µg/ml)
	820	1,650	3,300	6,580	13,160		
	Inhibition zone diameter (mm)						
GTE	0 ± 0	7 ± 2.3b	13 ± 1.2c	17 ± 0.58^d^	22 ± 0.58^d^	64	64
F1	0 ± 0	0 ± 0.0a	6 ± 0.0b	12 ± 0.58^c^	17 ± 1.00^c^	128	128
F2	0 ± 0	6 ± 0.7b	13 ± 3.2c	17 ± 1.15^d^	24 ± 0.58^d^	128	128
F3	0 ± 0	0 ± 0a	4 ± 3.5b	8 ± 2.31^b^	14 ± 0.00^c^	128	128
F4	0 ± 0	0 ± 0a	0 ± 0.0a	2 ± 3.46^a^	7 ± 2.31^b^	256	256
Ellagic acid	0 ± 0	0 ± 0a	0 ± 0.0a	0 ± 0.00^a^	0 ± 0.00^a^	>128	>128
Amoxicillin^a^	Nt	Nt	Nt	Nt	70 ± 1.5^e^	≤0.0625	≤0.0625
Metronidazole^a^	Nt	Nt	Nt	Nt	0 ± 0.0^a^	32	32

MIC: minimum inhibitory concentration.

MBC: minimum bactericidal concentration.

^a^ The analyzed concentration for amoxicillin was 200 μg/ml (10 μg/well) and for metronidazole was 100 μg/ml (5 μg/well).

Nt: not tested.

Values with different letters **(**a, b, c, d, e, f**)** are significantly different for the same concentration of *G. tinctoria* extract (*p* ≤ 0.05). Statistical analysis was made with ANOVA followed by Tukey's test.

**TABLE 4:** Well diffusion assay, MIC, and MBC for *H. pylori* strain J99 and inhibition upon ureases from Jack Bean and *H. pylori*.

Extract	*H. pylori* J99 strain								
	Concentration (µg/ml)					MIC (µg/ml)	MBC (µg/ml)	IC_50_ (µg/ml) Jack Bean	IC_50_ (µg/ml) *H. pylori*
	820	1,650	3,300	6,580	13,160^a^				
	Inhibition zone diameter (mm)								
GTE	0.0 ± 0.0	6.1 ± 0.6^b^	10.0 ± 1.5^c^	17.1 ± 0.0^d^	24.3 ± 0.0^e^	32	32	20.6	52.4
F1	0.0 ± 0.0	6.2 ± 0.0^b^	10.0 ± 0.6^b,c^	14.3 ± 0.6^c^	17.1 ± 0.6^d^	32	32	26.2	26.4
F2	0.0 ± 0.0	7.1 ± 0.6^b^	12.0 ± 3.2^c^	20.1 ± 2.0^e^	25.2 ± 1.0^e^	64	64	19.4	21.0
F3	0.0 ± 0.0	0.0 ± 0.0^a^	6.0 ± 0.0^b^	11.1 ± 0.6^b^	14.3 ± 0.6^c^	64	64	21.3	13.5
F4	0.0 ± 0.0	0.0 ± 0.0^a^	0.0 ± 0.0^a^	0.0 ± 0.0^a^	6.1 ± 5.5^b^	64	64	35.6	19.6
Ellagic acid	0.0 ± 0.0	0.0 ± 0.0^a^	0.0 ± 0.0^a^	0.0 ± 0^a^	0.0 ± 0.0^a^	>128	>128	—	—
Amoxicillin	Nt	Nt	Nt	Nt	67.2 ± 1.5^f^	≤0.0625	≤0.0625	—	—
Metronidazole	Nt	Nt	Nt	Nt	0.0 ± 0.0^a^	0.125	0.125	—	—
AHA	Nt	Nt	Nt	Nt	Nt	345	345	4.57	6.02

MIC: minimum inhibitory concentration.

MBC: minimum bactericidal concentration.

^a^ The analyzed concentration for amoxicillin was 200 μg/ml (10 μg/well) and for metronidazole was 100 μg/ml (5 μg/well).

AHA: acetohydroxamic acid in μg/mL.

Nt: not tested.

Values with different letters **(**a, b, c, d, e, f**)** are significantly different for the same concentration of *G. tinctoria* extract (*p* ≤ 0.05). Statistical analysis was made with ANOVA followed by Tukey's test.

## Conclusion

In the present report, thirty-five metabolites were detected using UHPLC-Q-Orbitrap-ESI-MS-MS for the first time in edible petioles of the native *G. tinctoria*. Furthermore, to the best of our knowledge, this is the first study of the anti-*H. pylori* activity of this plant. The results of the present work suggest that this edible plant has potential as antimicrobial agent because it acts upon the cellular wall not only causing lysis of bacterial cells but also inhibiting its urease enzyme. Both mechanisms can block the colonization of gastric mucosa and eventually ameliorate further infection processes. Due to its high anti-*H. pylori* effects, future *in vitro* and *in vivo* studies on *G. tinctoria* are planned.

## Data Availability

The raw data supporting the conclusions of this article will be made available by the authors, without undue reservation, to any qualified researcher.
