# Supplementary material for: Chilean Rhubarb, Gunnera tinctoria (Molina) Mirb. (Gunneraceae): UHPLC-ESI-Orbitrap-MS Profiling of Aqueous Extract and its Anti-Helicobacter pylori Activity
Source: Front Pharmacol. 2021 Jan 27;11:583961. doi: 10.3389/fphar.2020.583961 (PMC7941271; doi:10.3389/fphar.2020.583961)
Supplement: Supplementary file 1 [file datasheet1.pdf]

Supplementary Material

Chilean Rhubarb, *Gunnera tinctoria* (MOL.) MIRB. (Gunneraceae): UHPLC-ESI-ORBITRAP-MS profiling of aqueous extract and Its Anti-*Helicobacter pylori* Activity

Sonja Hebel Gerber[1, 2], Apolinaria García Cancino[2], Angélica Urbina[3], Mario J. Simirgiotis[4], Javier Echeverría [5*]; Luis Bustamante Salazar[6], Katia Sáez Carrillo[7], Julio Alarcón[8] and Edgar Pastene Navarrete[1, 8*]

[1] Laboratorio de Farmacognosia, Departamento de Farmacia, Facultad de Farmacia, Universidad de Concepción, Concepción, Chile.
[2] Laboratorio de Patogenicidad Bacteriana, Departamento de Microbiología, Facultad de Ciencias Biológicas, Universidad de Concepción, Concepción, Chile.
[3] Departamento de Producción Vegetal, Facultad de Agronomía, Universidad de Concepción, Chillán, Chile.
[4] Instituto de Farmacia, Facultad de Ciencias, Universidad Austral de Chile, Campus Isla Teja, Valdivia, Chile.
[5] Departamento de Ciencias del Ambiente, Facultad de Química y Biología, Universidad de Santiago de Chile, Santiago, Chile.
[6] Departamento de Análisis Instrumental, Facultad de Farmacia, Universidad de Concepción, Concepción, Chile.
[7] Facultad de Ciencias Físicas y Matemáticas, Universidad de Concepción, Concepción, Chile.
[8] Laboratorio de Síntesis y Biotransformación de Productos Naturales, Departamento de Ciencias Básicas, Universidad de Bío-Bío, Chillán, Chile.

*** Correspondence:**
edgar.pastene@gmail.com (E.P.); javier.echeverriam@usach.cl (J.E.)

Keywords: **Rhubarb; Mapuche food; Metabolomics; *Gunnera*, Pangue, Nalca; HPLC-MS; Orbitrap; *Helicobacter pylori.***

SUPLEMENTARY FIGURES

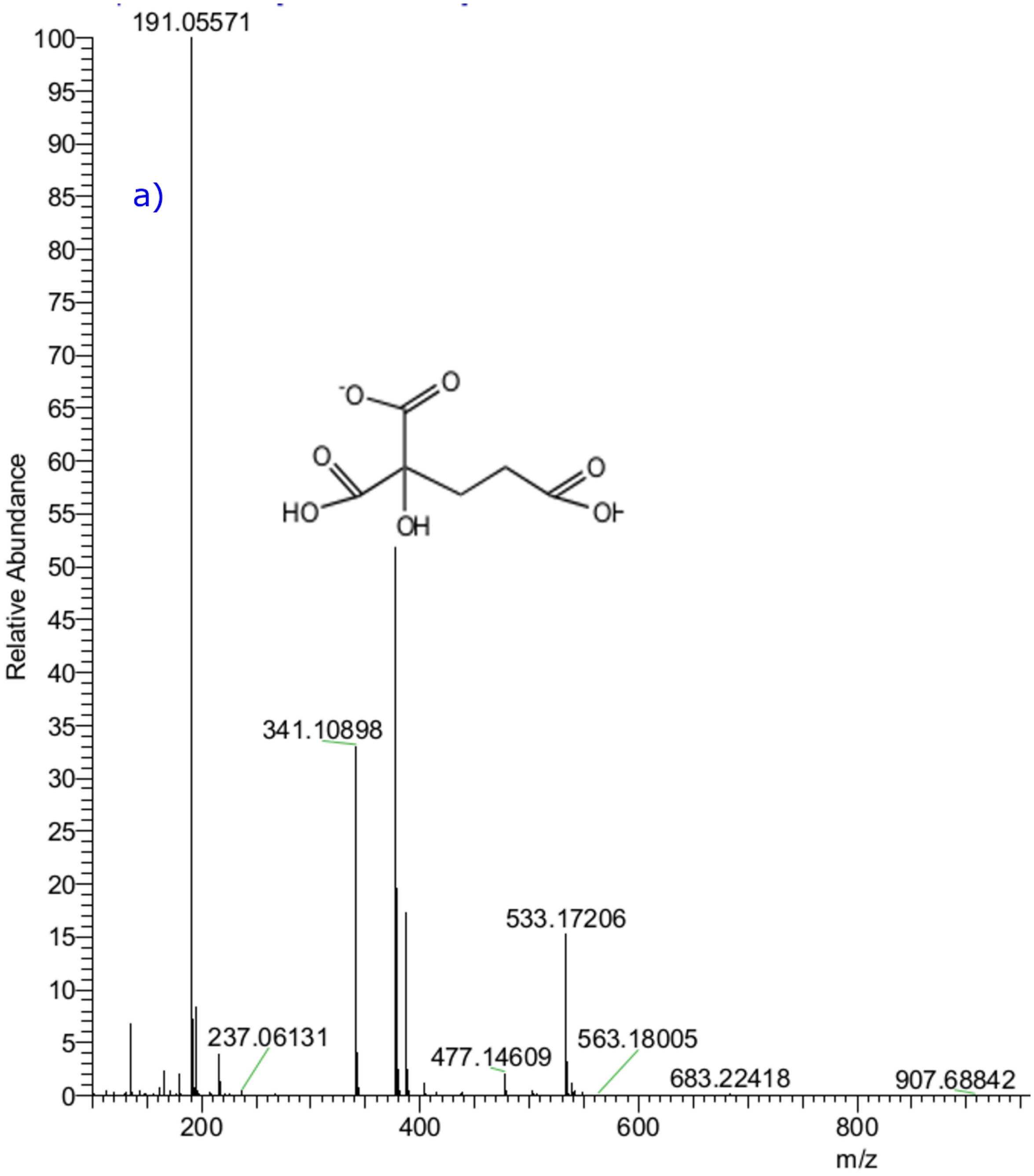

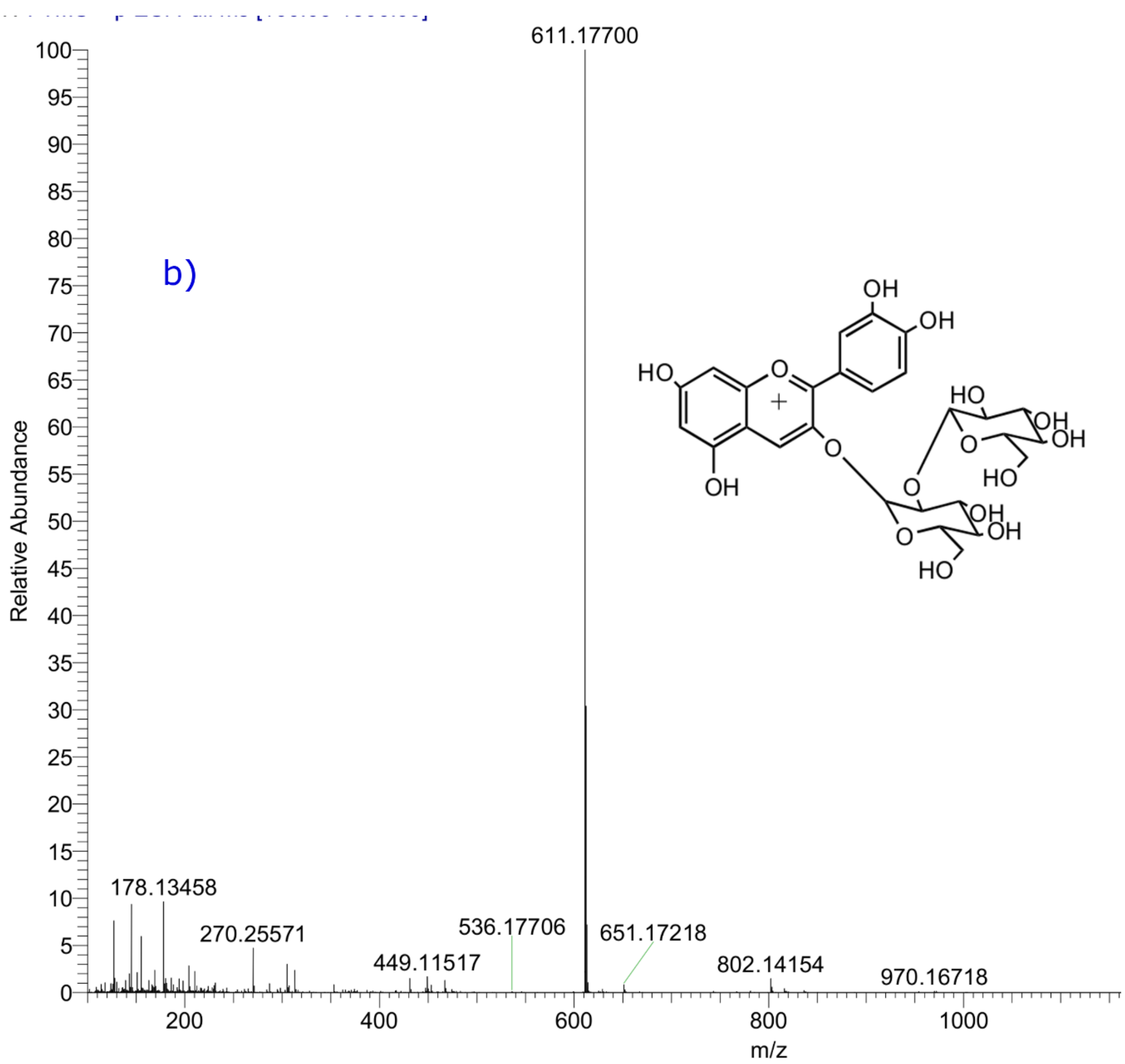
611.17700
b)
Relative Abundance
178.13458
270.25571
449.11517
536.17706
651.17218
802.14154
970.16718
m/z
HO
OH
OH
O
+
O
HO
OH
OH
HO
O
O
HO
OH
OH
O
HO
100
95
90
85
80
75
70
65
60
55
50
45
40
35
30
25
20
15
10
5
0
200
400
600
800
1000

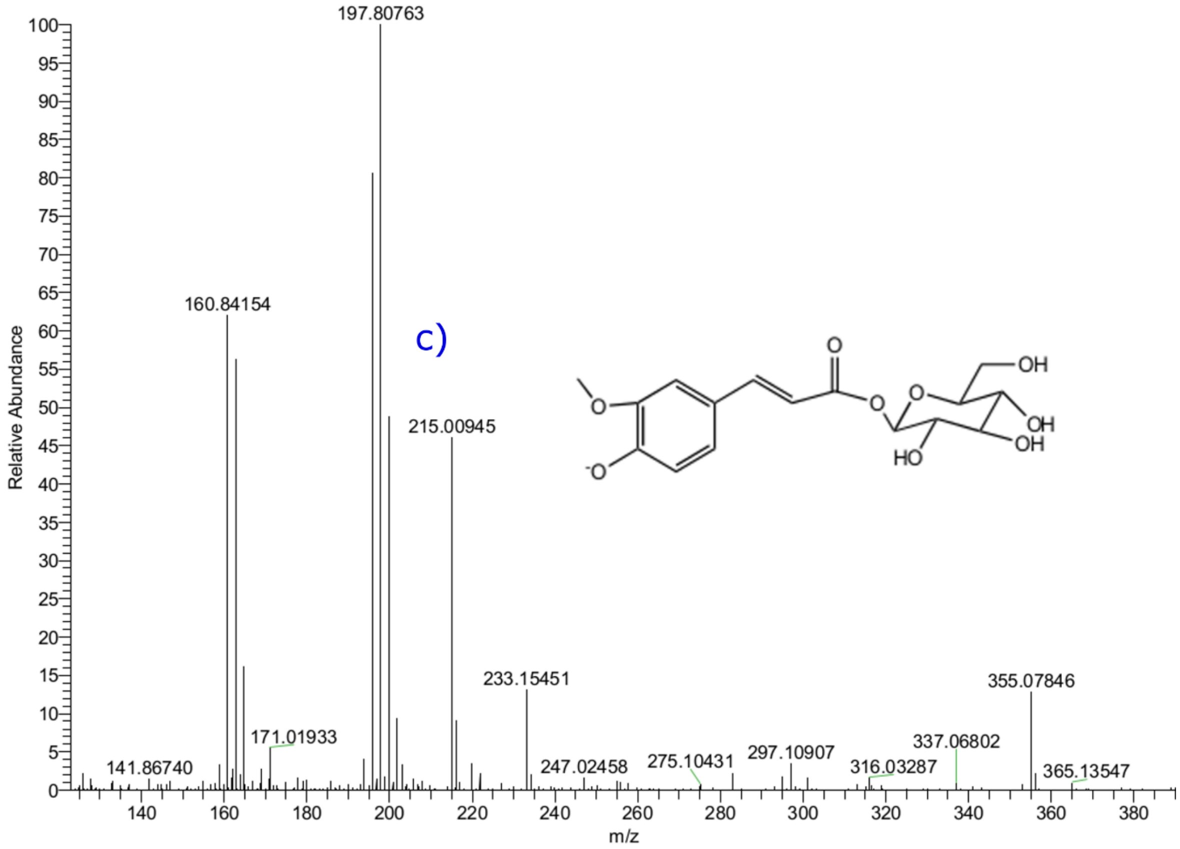
197.80763
160.84154
c)
215.00945
233.15451
355.07846
171.01933
141.86740
247.02458
275.10431
297.10907
316.03287
337.06802
365.13547
Relative Abundance
m/z
O
OH
OH
OH
HO

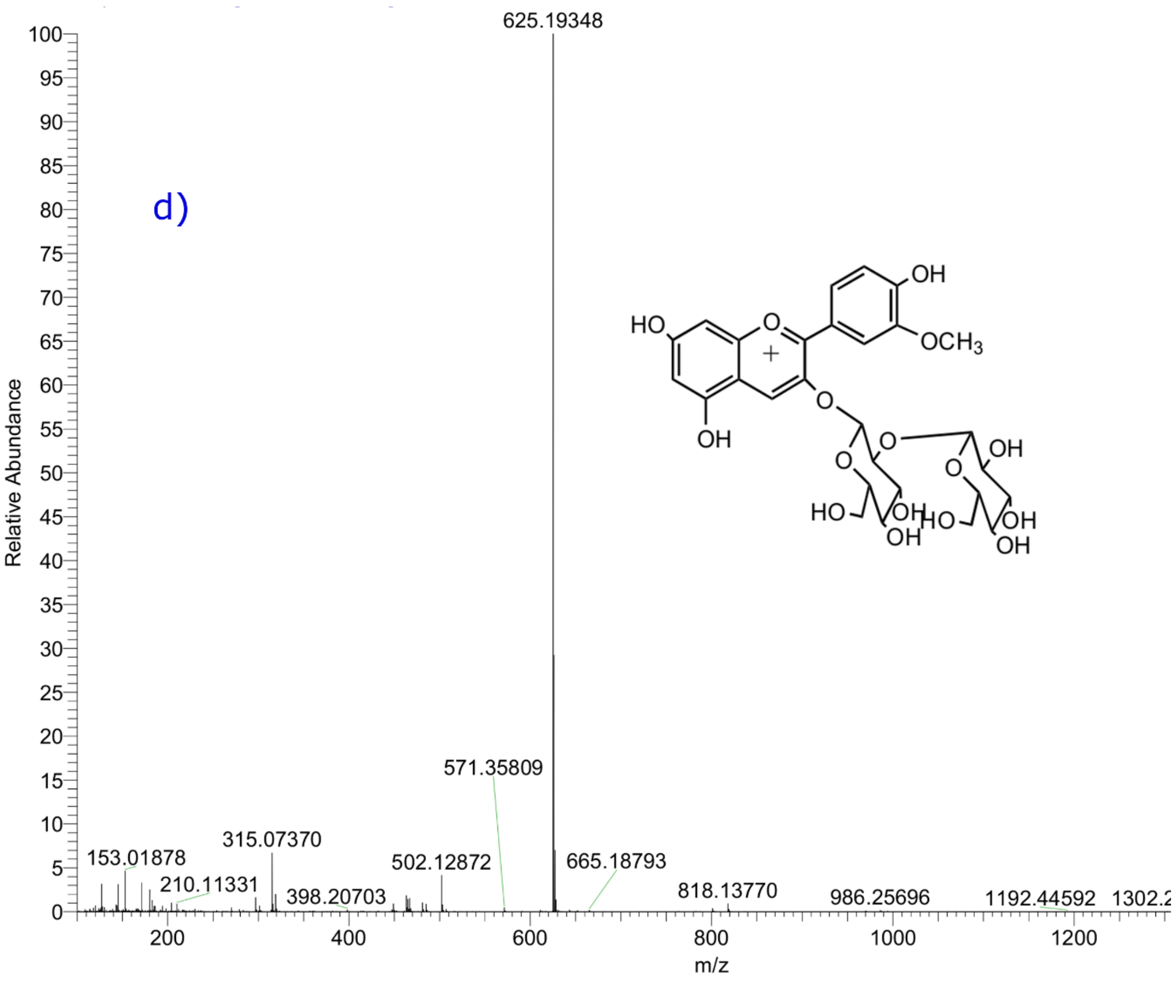
625.19348
d)
571.35809
315.07370
153.01878
502.12872
665.18793
210.11331
398.20703
818.13770
986.25696
1192.44592
1302.2
Relative Abundance
m/z
HO
OH
OCH3
OH
O
HO
OH
OH
HO
OH
OH
OH

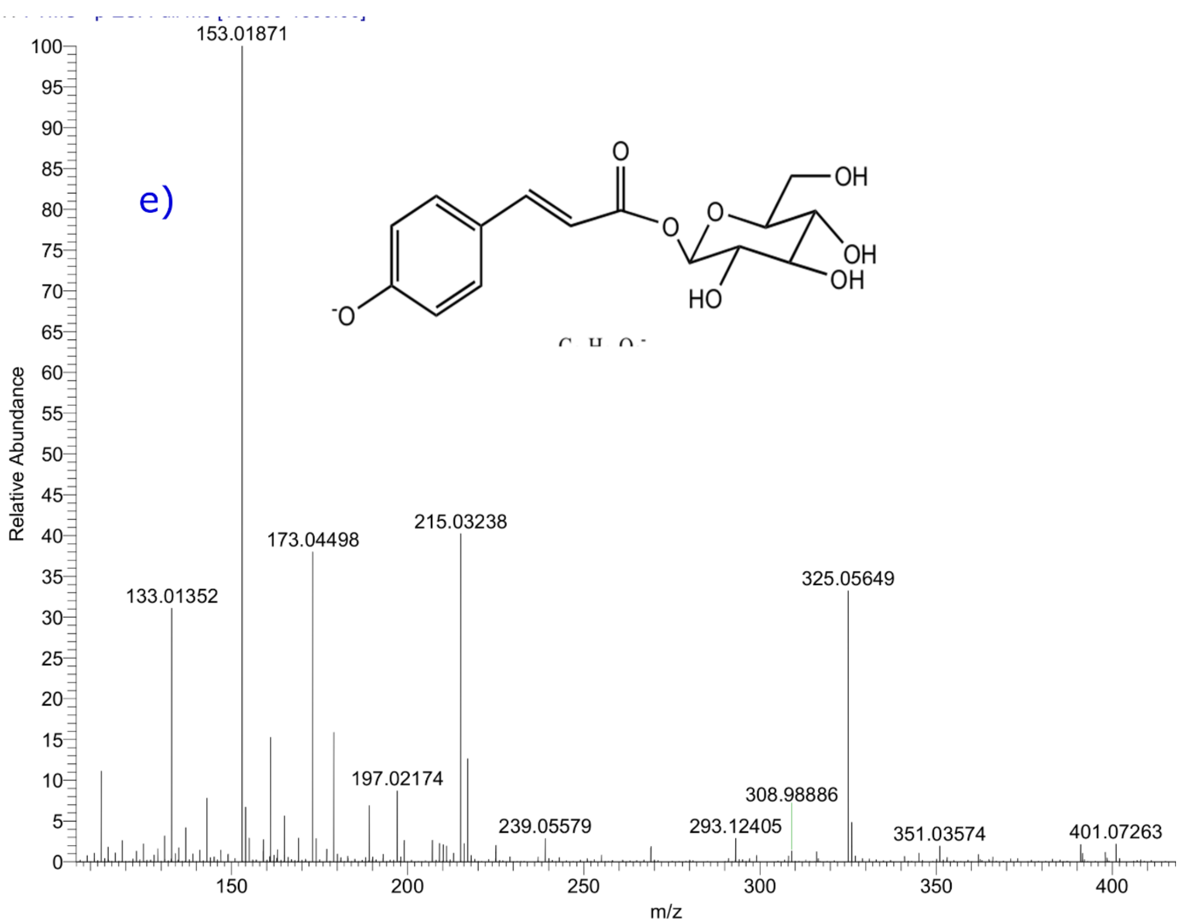
e)
153.01871
133.01352
173.04498
215.03238
325.05649
197.02174
239.05579
293.12405
308.98886
351.03574
401.07263
Relative Abundance
m/z
O
OH
OH
OH
HO
-O

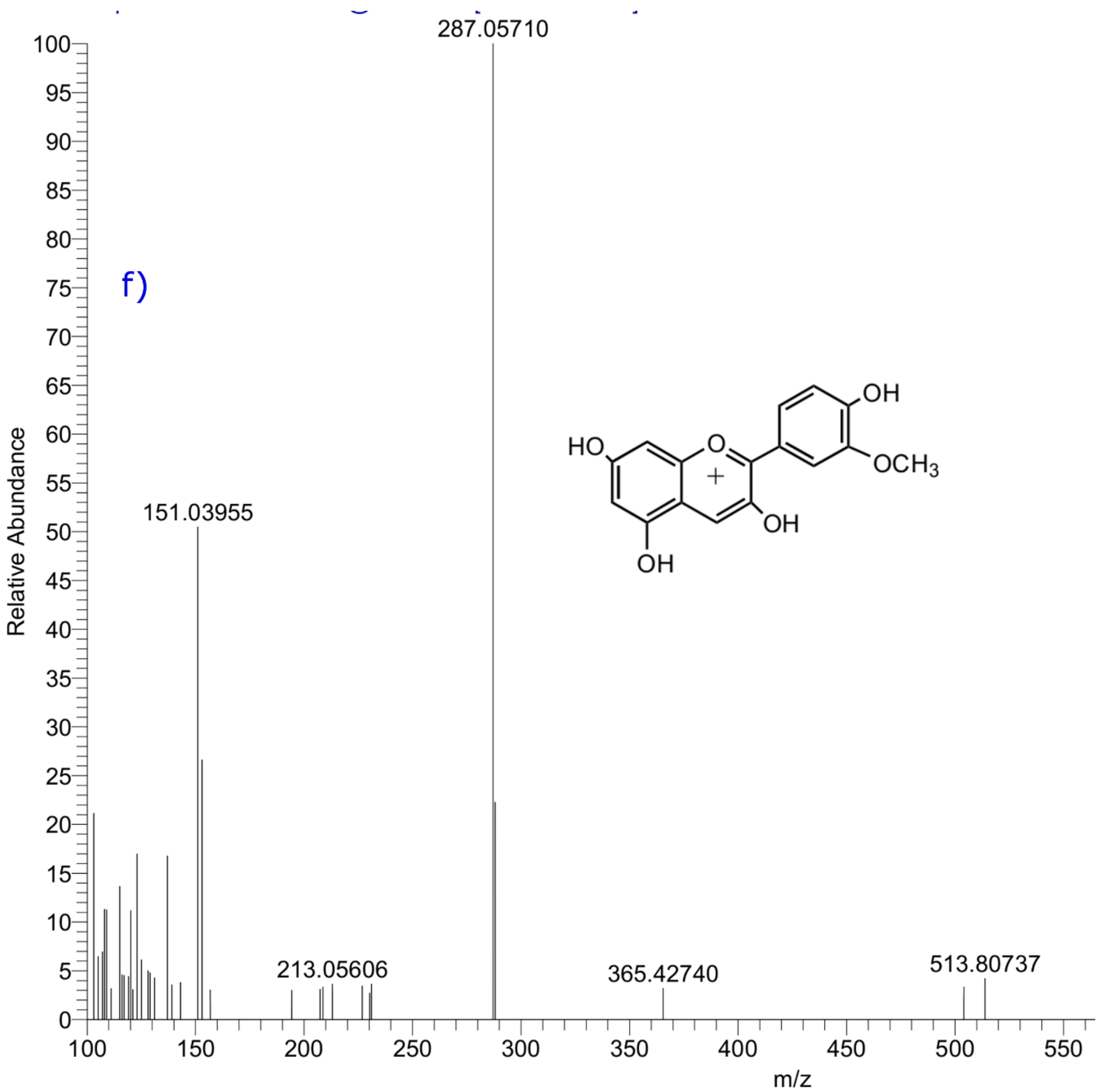
f)
287.05710
151.03955
213.05606
365.42740
513.80737
Relative Abundance
m/z
HO
OH
OH
OH
OCH_3
O
+

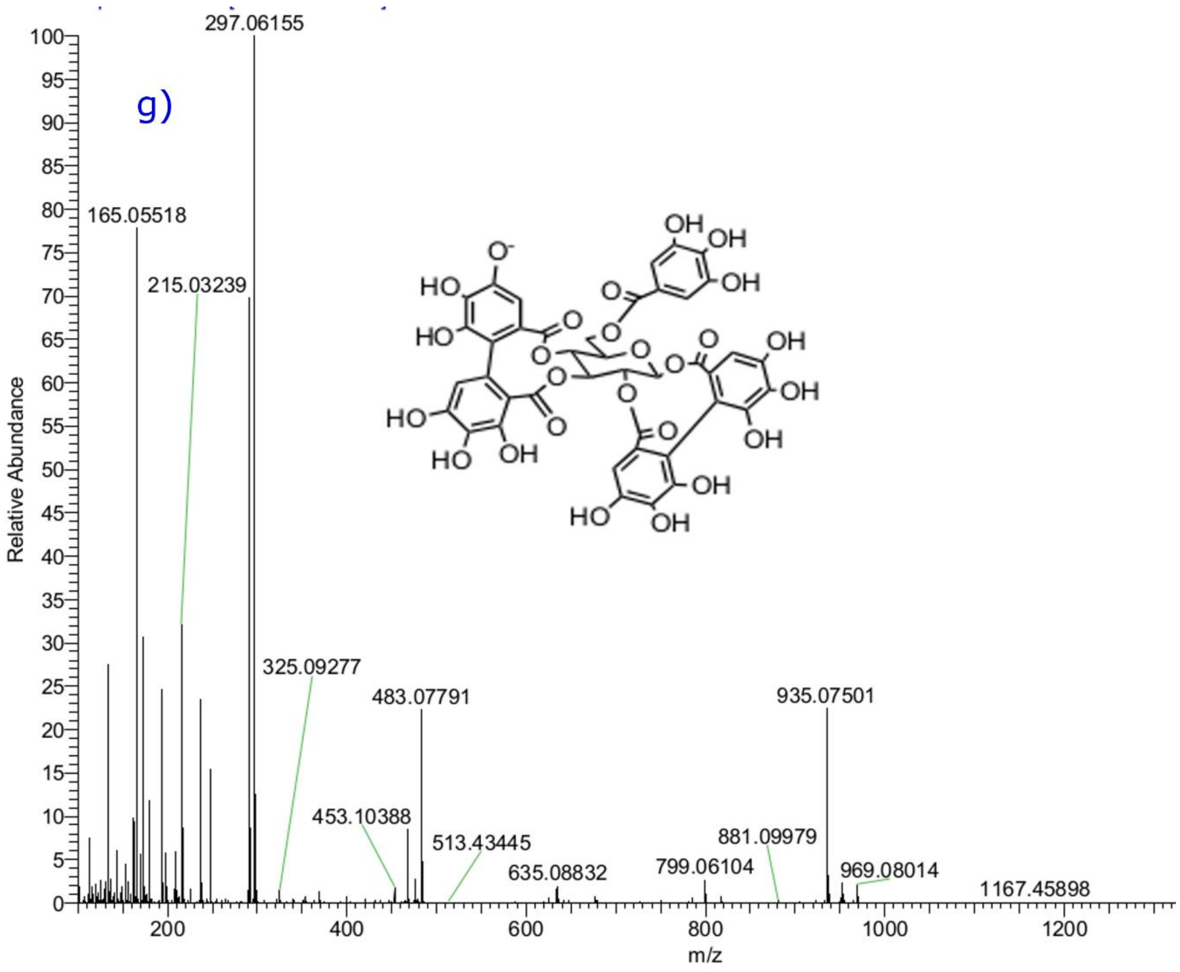
g)
297.06155
165.05518
215.03239
325.09277
483.07791
935.07501
453.10388
513.43445
635.08832
799.06104
881.09979
969.08014
1167.45898
Relative Abundance
m/z

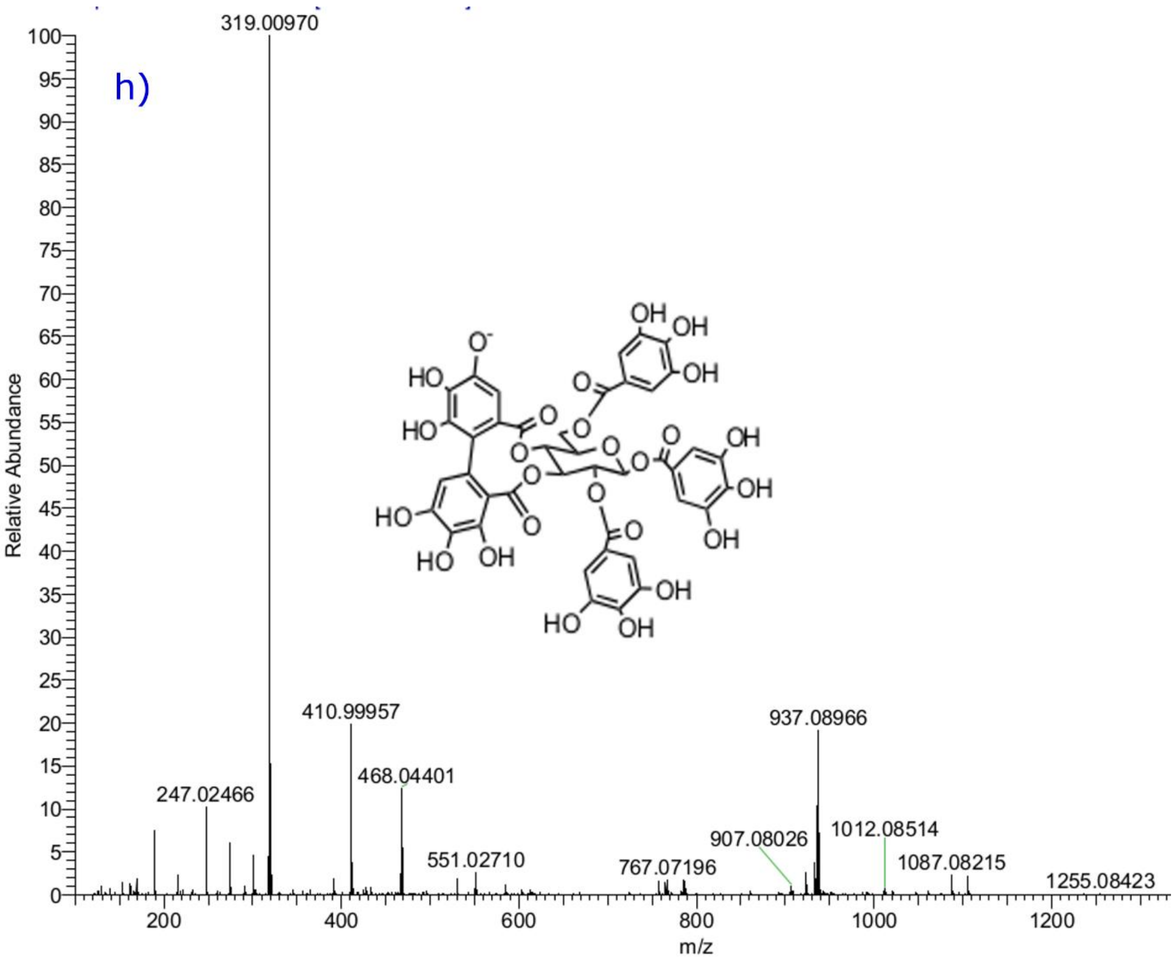
h)
319.00970
410.99957
468.04401
247.02466
551.02710
767.07196
907.08026
937.08966
1012.08514
1087.08215
1255.08423
Relative Abundance
m/z

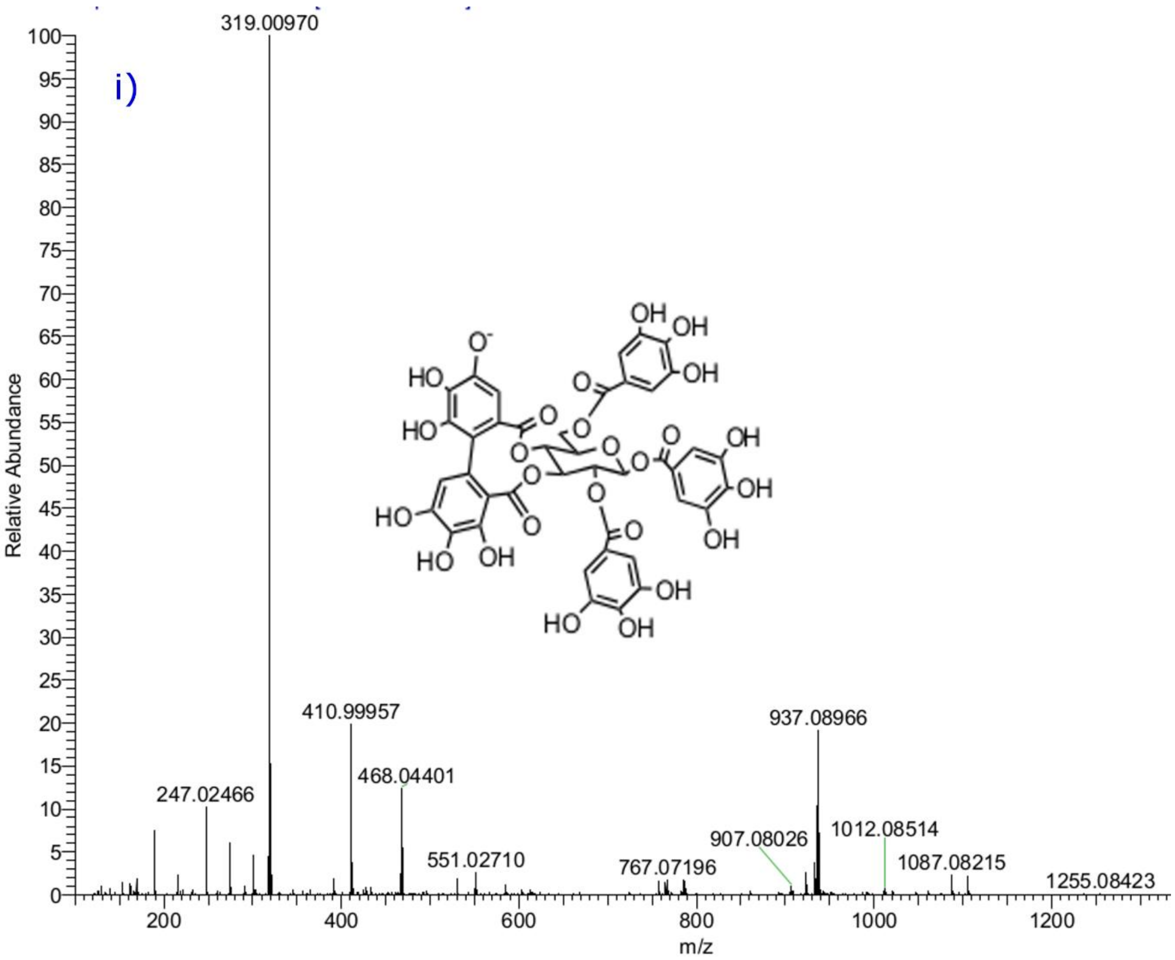

i)
319.00970
410.99957
468.04401
247.02466
551.02710
767.07196
907.08026
937.08966
1012.08514
1087.08215
1255.08423
Relative Abundance
m/z

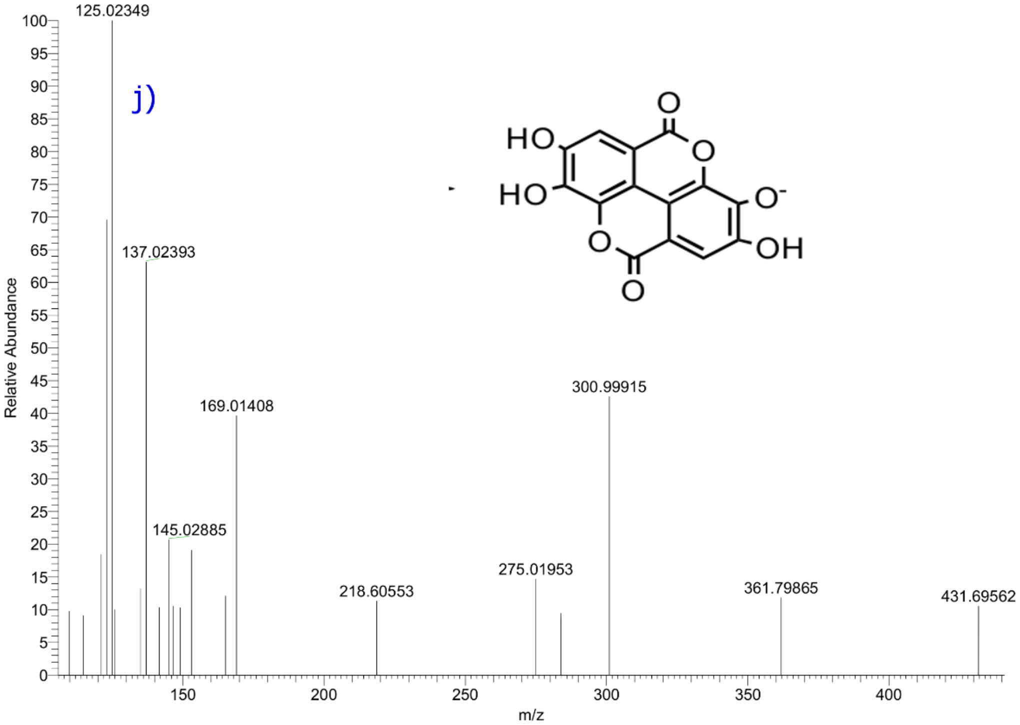

j)
125.02349
137.02393
169.01408
145.02885
218.60553
275.01953
300.99915
361.79865
431.69562
Relative Abundance
m/z

Supplementary Figure 1. Figures (a-k): Full MS spectra and structures of peaks 1, 7-10, 18, 23-26 and 28, respectively.

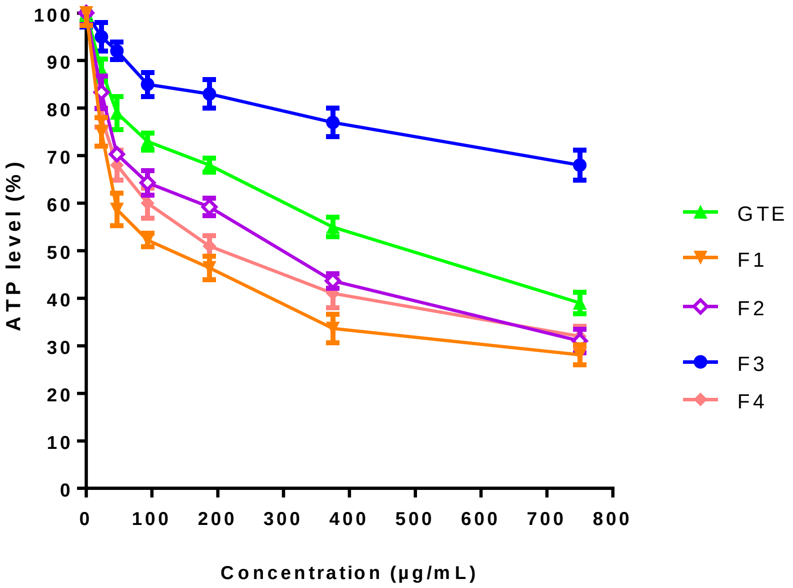

Supplementary Figure 2. GTE and its CPC fractions effect upon the *H. pylori* ATP levels. CFU/mL was incubated in the presence of samples (0-1800 µg/mL), under microaerobic conditions during 1 h. ATP levels were analyzed by chemiluminiscence as described in Materials and Methods section.
